# Small Angle Scattering Techniques on In Situ Adsorption Studies: A Comprehensive Review

**DOI:** 10.3390/ma19173614

**Published:** 2026-08-25

**Authors:** Ramonna I. Kosheleva, Maria Tsaroucha, Ioanna Xylouri, Konstantinos Pavlidis, Agni A. Moutzouroglou, Theodoros Markopoulos, Athanasios Ch. Mitropoulos

**Affiliations:** Hephaestus Lab, Department of Chemistry, Democritus University of Thrace, St. Lucas, 65404 Kavala, Greece

**Keywords:** small-angle X-ray scattering, small-angle neutron scattering, in situ SAS, operando SAS, time-resolved characterization, contrast variation, soft matter, carbon materials, biological systems, adsorption kinetics, contrast matching

## Abstract

In situ and operando small-angle X-ray and neutron scattering (SAXS and SANS) have become powerful techniques for studying adsorption processes because they provide real-time information that traditional ex situ methods cannot capture. This review examines the use of these techniques in four major material groups: soft matter and polymers, carbon-based materials, biological systems and ceramics. Unlike conventional before-and-after characterization, in situ methods allow researchers to follow structural changes during adsorption as they happen, revealing adsorption kinetics, intermediate states and pore-filling mechanisms. Operando approaches further connect nanoscale structural evolution with overall adsorption performance, leading to deeper mechanistic understanding. Important developments include monitoring structural rearrangements in polymers during adsorption, studying sodium storage mechanisms in hard carbon anodes using combined small- and wide-angle neutron scattering, observing protein conformational changes at hydrated interfaces, and identifying organic functional groups in mesoporous ceramics in real time. SANS contrast variation, especially through hydrogen/deuterium substitution, allows selective visualization of different components in complex systems, while synchrotron SAXS enables kinetic studies with millisecond time resolution. Despite these advantages, several challenges remain, including model-dependent interpretation of scattering data, difficulties in separating real structural changes from contrast-related artifacts, limitations in accessible timescales and the technical complexity of isotopic labeling. Future research is expected to focus on improved contrast methods, machine learning-assisted data analysis, multimodal approaches combining scattering with spectroscopy or microscopy and the application of these techniques to more complex and disordered adsorbents such as activated carbons, coals and bio-based materials. Overall, this review summarizes current methodologies, compares adsorption-related structural changes across different materials and highlights both current limitations and future opportunities for in situ and operando SAS studies in adsorption research.

## 1. Introduction

The scientific study of adsorption dates back to the 18th century with Scheele’s first observations about gas uptake by charcoal, which led to Langmuir’s kinetic theory of monolayer adsorption in 1916 [1]. Since then, adsorption has lain at the heart of various industrial processes. Adsorption is considered critical for researching advanced materials as it forms the basis of chemical and physical processes in modern technology and environmental sustainability. From gas separation and storage [2] to heterogeneous catalysis [3] and water purification [4], the ability of a surface to selectively bind molecules and thus cause the enrichment of molecules in solid-fluid interfaces determines efficiency, selectivity and capacity. Beyond these core applications, adsorption also plays a vital role in carbon capture and sequestration [5], drug delivery [6] and chromatographic separation of pharmaceuticals and fine chemicals [7].

During the latest decades, advanced porous materials, whose adsorption properties are the result of their nanoscale structure, such as metal–organic frameworks (MOFs), covalent organic frameworks (COFs), mesoporous silicas, activated carbons and polymeric networks, have been developed to meet the demand for ever-higher performance in adsorption processes. For instance, hydrogen-bonded organic frameworks (HOFs) have emerged as promising platforms for the selective separation of noble gases and light hydrocarbons [8], while supramolecular macrocycles are increasingly applied to remove organic and inorganic pollutants from water [9]. Understanding in full depth how and where adsorbate molecules reside, and the way they reorganize and how the host framework responds during the procedure constitutes a prerequisite for material design.

Over the years of research, especially in adsorption processes, ex situ characterization has been widely used. Despite the various applications that are well established [10], ex situ characterization provides only static snapshots as the adsorbent is measured only two times, before and after exposure to an adsorptive [11,12]. As a result, in the period between those states that is not observed, the material may undergo reversible structural changes, pore filling transitions, or adsorbate rearrangements that are completely missed [13]. At the same time, there is a limitation linked with ex situ characterization, as these methods cannot capture kinetic pathways, metastable intermediates, or the coupling between adsorption and the mechanical deformation of the host. For this exact reason, research has been focused on the development of in situ characterization, in which the material is observed during exposure to the adsorptive under controlled conditions [14]. Furthermore, operando techniques have been taking center stage as the material is measured concurrently with the structural characterization [15,16]. Taking into consideration that operando approaches are especially important because of their correlation of structural events happening at the nanoscale with macroscopic adsorption metrics [17], it is significant to lead research into that pathway. In situ and operando techniques have the potential to enable mechanistic rather than merely descriptive results and conclusions [18].

There are a variety of in situ and operando techniques such as vibrational spectroscopy, nuclear magnetic resonance and electron microscopy [19,20,21], although small-angle scattering techniques stand out for their unique combination of features [22]. Both small-angle X-ray scattering (SAXS) and small-angle neutron scattering (SANS) probe length scales from approximately 1 nm to several hundred nanometers as they cover precisely the pore size, particle dimensions and fractal features relevant to most engineered adsorbents [23,24]. These techniques are statistical and probe large ensembles of pores simultaneously, providing representative information about the pore network [25]. Small-angle light scattering (SALS), particularly when combined with rheology, provides real-time access to mesoscale structural evolution and phase transitions in soft-matter systems under flow, as evidenced by its ability to resolve changes in scattering patterns associated with lamellar and multilamellar vesicle structures. However, it does not provide direct quantitative information on adsorption and is therefore best interpreted as a structural rather than compositional probe [26]. This fact leads to the removal of the risk of bias of local imaging methods [27,28]. The benefits of these techniques are numerous, with some of them being that they are compatible with realistic sample environments such as variable temperature and pressure, gases and liquids, and at the same time, they can be time-resolved to follow adsorption kinetics for a long period of time (seconds to hours) [29,30].

One of the most significant advantages of small angle scattering techniques is that they offer complementary contrast mechanisms [31]. In particular, SAXS has the capability to exploit the differences in the electron density at laboratory and synchrotron sources, creating contrast variation and therefore enabling fast acquisition [32]. On the other hand, SANS exploits nuclear scattering length density differences, which can be used to determine the degree of closed porosity [33]. Also, it can be tuned via hydrogen/deuterium isotopic substitution, which is extremely powerful as it is used for contrast matching the adsorbent or the adsorbate; thereby, it can reveal otherwise hidden components [34,35]. Applying these mechanisms in situ and operando, it is possible to resolve problems such as whether the capillary condensation is proceeding via a continuous or discontinuous meniscus [36] or if the adsorbates form layers or clusters [37]. Consequently, it is crucial to examine the role of SAS techniques for the aforementioned reasons.

This work aims to provide a coherent assessment of the application of in situ and operando SAXS and SANS to the study of adsorption phenomena. This review is focused on the latest experiments where small scattering data were collected during adsorption (in situ) or along with adsorption performance measurements (operando) in order to specifically study the procedure. It is necessary to distinguish these works from the classic ex situ or post-mortem studies. Emphasis is placed on four categories of materials: (i) ceramics, (ii) soft matter and polymer systems, (iii) carbon-based materials and (iv) biological systems. Nevertheless, SAXS and SANS are widely discussed in the literature; their combined and complementary in situ and operando use is underdeveloped, but it yields insights unattainable by classic use of the SAS techniques. The purpose of this review is to develop a coherent framework linking experimental design, contrast strategies and quantitative adsorption models. Challenges persist in data interpretation, particularly in complex, multicomponent systems where multiple scattering contributions overlap. For this reason, we intended to clarify three different objectives. Firstly, to consolidate best methodological practices; secondly, to compare the structural signature of the adsorption phenomenon across different material classes; and lastly, to identify the current limitations and the emerging opportunities of the use of these techniques for in situ and operando studies of adsorption. This review is intended both for simply seeking an entry point into in situ scattering for adsorption and for looking at critical evaluation of recent advances and future directions.

## 2. Fundamentals of Small-Angle Scattering Techniques

### 2.1. Small-Angle Scattering Techniques

**Figure 1:**
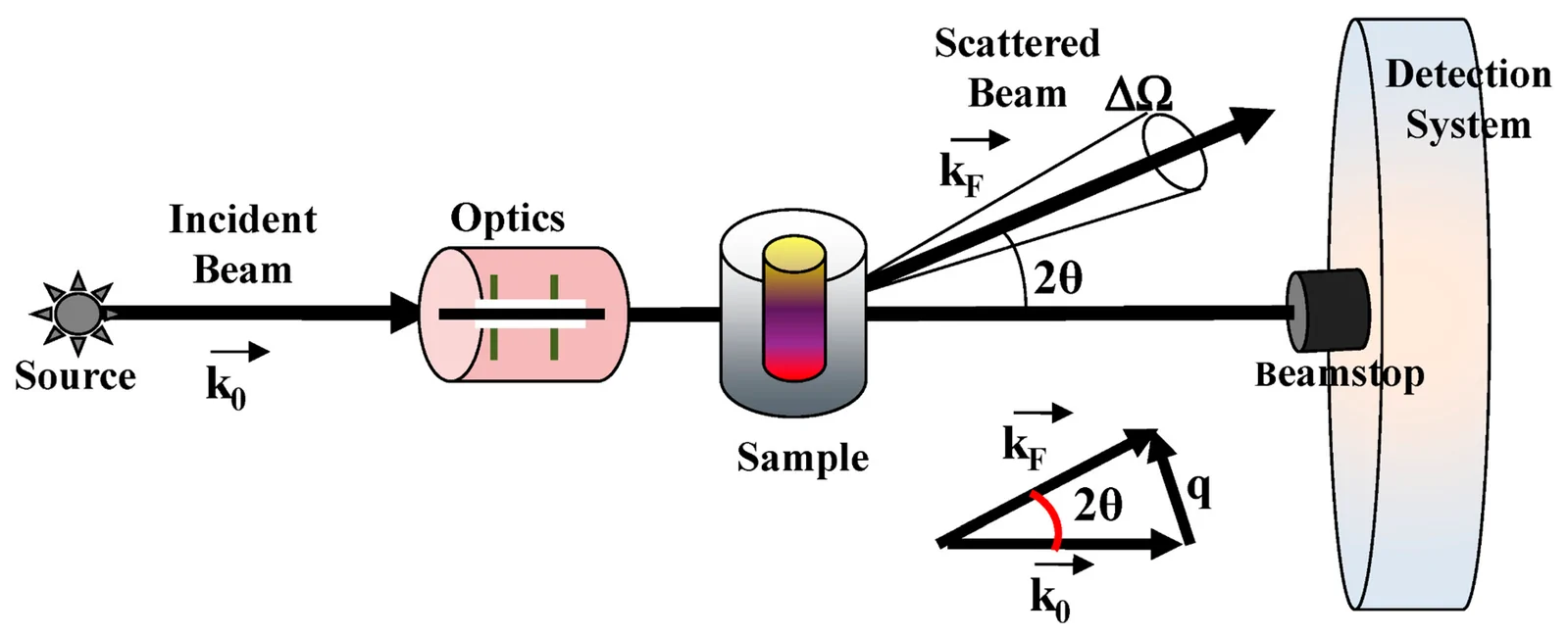
In a typical small-angle scattering (SAS) experiment, a beam of neutrons or X-rays is directed at a sample. The beam interacts with the material and scatters at an angle denoted as 2θ. This scattered radiation is then captured by a suitable detector; the scattering factor, denoted as q, is defined by the difference between the scattered (k_F_) and incident (k_0_) wave vectors [45].

Small-Angle Scattering (SAS) techniques are a class of non-destructive, non-intrusive analytical techniques used to probe structural features of materials on the nanoscale, typically in the range of approximately 1–100 nm, by analyzing how radiation is elastically scattered after interacting with a sample [38,39,40,41]. These techniques include SAXS, SANS and small-angle light scattering (SALS), using X-rays, neutrons or visible light, respectively, allowing access to structural information across a wide range of materials and length scales (Figure 1). Depending on the experimental setup, SAS is even able to extend its sensitivity towards larger structures approaching the submicron scale, up to 1 μm in some cases, making it suitable for hierarchical and multiscale systems [38,40].

The core principle of SAS is based on measuring the scattering intensity produced by spatial variations in electron density for SAXS or nuclear density for SANS, which arise from structural differences between phases in a material [42]. These density fluctuations create contrast, which is the fundamental driving factor that determines the strength and nature of the scattering signal and ultimately enables structural interpretation. From the measured scattering patterns, key structural parameters can be extracted, including particle size and size distributions, particle shape, internal density variations, aggregation state and surface-to-volume characteristics [31,39,40,41]. A central mathematical framework of SAS Equation (1) involves separating the scattering intensity into contributions from the form factor, which describes the geometry and internal structure of individual particles, and the structure factor, which accounts for spatial correlations and interactions between particles:

(1) I(q)=NP(q) S(q)

where *N* is the number of particles in the scattering volume, *P*(*q*) is the form factor containing shape information, and *S*(*q*) is the structure factor displaying the spatial arrangement of the particles [31,40,41].

However, this classical product representation is strictly valid only for idealized, monodisperse, spherically symmetric systems where the spatial positions of the particles are uncorrelated with their sizes or shapes. In real physical systems, polydispersity and non-spherical geometries are highly prevalent, meaning that the overall parameters and the measured one-dimensional scattering profile *I*(*q*) do not represent a single physical state but a weighted average over all the molecular ensembles present in the solution [43]. For discrete mixtures of *K* components, the total scattering intensity is described as a linear combination of the individual contributions of each species:

(2) $$I\left(s\right)=\sum_{k=1}^{K}\eta k\;Ik\;(s)$$

where *η_k_* is the volume fraction of species.

If the system instead exhibits continuous polydispersity (as seen in highly flexible macromolecules or complex colloidal nanoaggregates), the size distribution must be modeled continuously [44]. Under these conditions, the average form factor term <*P*^2^(*q*)> is integrated over a size distribution function *f*(*R*) and orientation angles, yielding:

(3) $$<P^{2}(q)>\;=\int_{0}^{\infty }f(R)\;dR\;\int_{0}^{\pi /2}{\left[P(q,R,a)\right]}^{2\;}sin\;a\;da$$

where *f*(*R*) represents a size distribution function (such as a log-normal distribution), and α is the orientation angle between the normal of the anisotropic particle and the scattering vector *q* to ensure proper orientational averaging.

Furthermore, when interparticle interactions are significant in concentrated or interacting polydisperse regimes, spatial correlations depend on the specific sizes of neighboring particles. To describe the coherent macroscopic scattering cross-section under these conditions, the decoupling approximation is introduced. The physical assumption underlying this approximation is that the size and orientation of any given particle are entirely uncorrelated with its relative spatial position in the ensemble [44]. Under the decoupling approximation, the total coherent scattering intensity is written as:

(4) $$I(q)=\;N_{p}<P^{2}(q)>S^{\prime }(q)$$

where *N_p_* = *φ*/*V_p_* is the average particle number density defined by the volume fraction *φ* and the average particle volume *V_p_*. Here, *S*′ is the effective structure factor, which accounts for both the interparticle interactions and the decoupling of size/shape from spatial distribution. The effective structure factor *S*′(*q*) is related to the ideal structure factor *S*(*q*), which describes center-of-mass correlations of equivalent spheres, via:

(5) $$S^{\prime }(q)=1+\beta (q)[S(q)-1]$$

where the decoupling parameter *β*(*q*) acts as a *q*-dependent scaling factor defined by:

(6) $$\beta (q)=\frac{{<P(q)>}^{2}}{<P^{2}(q)>}$$

The value of the decoupling parameter *β*(q) is strictly bounded between 0 and 1. For a perfectly monodisperse and spherically symmetric system *β*(*q*) = 1, which simplifies the expression back to the classic product representation *I*(*q*) = *NP*(*q*)*S*(*q*). Conversely, when particles exhibit significant anisotropy or large size polydispersity, *β*(q) < 1, which systematically dampens the contribution of the ideal structure factor S(*q*) to the total scattered intensity [44].

The scattering vector magnitude *q* in these expressions is defined by the wavelength of the incident radiation and the scattering geometry:

(7) $$q=\;(\frac{4\pi }{\lambda })\;sin\;(\theta )$$

where *λ* is the wavelength of the incident beam, and 2*θ* is the angle of scatter. *I*(q) is the measured signal, which gives context on the length scale of the probing, *P*(*q*) is the form factor and contains shape information and *S*(*q*) is the structure factor, which shows the way the particles are arranged together [31,40,41].

Relative intensity variations between samples or within a scattering profile reflect combined changes in how many scattering objects are present, how strongly they scatter relative to the surrounding medium, and how they are organized spatially [41,46,47,48]. Even when particle shape remains unchanged, differences in concentration or scattering contrast can significantly alter the measured intensity scale, meaning that meaningful comparison of relative intensity requires consistent experimental conditions [46,47]. In dilute systems where interparticle interactions are negligible, the structure factor approaches unity and intensity primarily reflects single-particle scattering, allowing relative intensity to be interpreted mainly in terms of particle content and internal scattering strength. Across the scattering vector range, relative intensity variations also encode structural hierarchy, since low, intermediate, and high q regions correspond to different length scales such as large-scale morphology, particle size distributions and surface or sub-particle features. Furthermore, contrast effects, such as electron density differences in SAXS or neutron scattering length density differences in SANS, can selectively enhance or suppress specific contributions, meaning that relative intensity is highly sensitive to compositional tuning, including isotopic substitution in SANS or solvent contrast matching [49]. Because of this sensitivity, relative intensity is widely used for qualitative or semi-quantitative structural interpretation, such as identifying phase separation, adsorption effects, porosity or hierarchical self-assembly in complex materials [48]. However, relative intensity is not inherently on a physical scale, since it depends on experimental conditions, background subtraction and instrument response, which limits its use for direct quantitative comparison across different setups or studies [31,48].

By contrast, absolute intensity refers to fully calibrated scattering data in which instrumental factors such as beam flux, sample thickness, detector efficiency and geometry are accounted for, preserving the true proportionality between signal and physical scattering power [41,48]. On this absolute scale, intensity becomes directly proportional to physically meaningful quantities such as particle number density, volume fraction, and squared contrast, enabling quantitative determination of structural parameters rather than purely comparative analysis [41,46,48]. Thus, while relative intensity is essential for identifying structural trends and hierarchical organization, absolute intensity is required for rigorous quantitative interpretation of nanoscale structure and for comparing results across different experiments or techniques [31,41,46].

One of the major strengths of SANS compared to SAXS is its ability to exploit isotopic contrast variation, particularly through hydrogen/deuterium substitution, enabling selective highlighting or covering specific components in complex systems [42,43,44]. This capability is especially powerful in biological and soft-matter systems, where macromolecular complexes, multi-component assemblies and flexible structures can be studied under near-native solution conditions [31,41,50].

In materials science and porous media research, SAS is particularly valuable because it can probe pore structures ranging from mesopores to macropores, including mixed and hierarchical pore networks [38]. Importantly, SAS can detect both open and closed pores, something that many intrusive techniques, such as gas adsorption, cannot achieve, allowing a more complete understanding of internal structure and accessibility [42]. Beyond static characterization, modern SAS techniques also enable in situ and real-time monitoring of structural evolution during processes such as hydration, adsorption, phase transitions or thermal treatment [39,51]. The use of synchrotron radiation and advanced detectors has significantly improved temporal resolution and accessible scattering ranges, allowing dynamic processes to be observed with high precision.

In structural biology, SAS is widely used to study proteins, nucleic acids and their complexes in solution, providing low resolution but highly valuable information on shape, flexibility, oligomerisation state and conformational changes. SANS is particularly important to this field due to its sensitivity to hydrogen atoms and its ability to exploit contrast matching, which allows individual components of complex biomolecular systems to be selectively analyzed [31,41,50]. In addition, SAS is widely applied to polymers, colloids, nanocomposites, catalysts, glasses and cementitious systems, where it helps connect nanoscale structure to macroscopic material properties [38,39,40].

Because it requires minimal sample preparation and is compatible with solids, liquids and soft matter, SAS has become a highly versatile tool across chemistry, biology, physics and materials science. Overall, SAS techniques provide a unique combination of structural sensitivity, non-destructive measurements and multiscale applicability, making them essential for understanding both equilibrium structures and dynamic processes in complex systems [51,52].

#### 2.1.1. Small-Angle X-Ray Scattering

SAXS is widely applied to determine particle size and size distribution, particle shape and electron density variations in systems where structural features lie beyond the resolution of conventional X-ray diffraction methods [53,54,55,56,57]. The technique is based on elastic scattering of X-rays by electrons, where interference of scattered waves produces intensity patterns that can be related to structural information through the scattering vector q [53,55,58,59], as mentioned above.

SAXS is particularly valuable because it can be adapted to a wide range of experimental conditions, including synchrotron-based setups that offer high flux, excellent beam quality and tunable wavelengths, enabling time-resolved studies of dynamic processes such as phase transitions, crystallization and polymerisation [53,54,55]. Advanced beamline configurations, including long sample-to-detector distances, vacuum flight paths and area detectors, are essential for accurate low-angle measurements and improve signal quality [53,55,60].

In nanomaterials and colloidal systems, SAXS provides detailed information about nanoparticle morphology, spatial organization, interparticle distances and structural evolution during processes such as growth or annealing [54,56,57]. Surface-sensitive variants such as grazing-incidence SAXS (GI-SAXS) extend its capabilities to thin films and supported nanoparticles, allowing the analysis of in-plane and out-of-plane ordering and anisotropic structures [54]. In porous and heterogeneous materials, SAXS can resolve structural features such as pore size distribution and anisotropy, even enabling spatially resolved measurements using microbeam techniques [60,61]. USAXS (often combined with SAXS) has also been employed to probe adsorption-related structural evolution in heterogeneous materials, where adsorption or adsorbate–substrate interactions induce measurable changes in hierarchical organization across the nano- to submicron scale (~1 nm to a few µm). In such systems, adsorption of species such as AcOH in MOFs can lead to transitions from dense aggregates toward more open or hollow morphologies, accompanied by variations in low-q scattering slopes and fractal exponents that reflect changes in packing density and network reorganization [62]. Similarly, in catalyst ink systems, ionomer adsorption on Pt/C particles has been shown to systematically alter the agglomerate structure, with increasing adsorption corresponding to a reduction in fractal dimension from ~2 toward ~0.5, indicating a transition from a gel-like network to more dispersed structural units while leaving primary aggregates (~200 nm) largely unaffected [63]. More generally, these scattering techniques provide indirect access to adsorption-governed changes in pore connectivity, surface area, and hierarchical architecture, which control both adsorption site accessibility and transport behavior in porous materials [64].

SAXS is also used to investigate soft-matter systems such as polymers and biological assemblies, where it provides insights into radius of gyration, internal density distribution and conformational changes in solution [57,65]. It can distinguish different structural morphologies, such as globular, cylindrical, lamellar or fractal, based on scattering behavior across different q-ranges [57,60]. Additionally, periodic structures produce Bragg peaks, from which characteristic spacings between ordered features can be directly determined [60].

Despite its versatility, SAXS requires careful experimental conditions, including highly monodisperse and concentrated samples for reliable interpretation, as well as proper correction for background scattering and sample heterogeneity [53]. Heterogenous systems or the presence of interfering components can complicate data analysis and reduce accuracy [59].

#### 2.1.2. Synchrotron SAXS

Synchrotron radiation sources have the capability to provide extremely high brilliance and collimation, thus being widely used in small-angle scattering techniques and especially SAXS in which high temporal resolution is required. Structural biology is one of the fields in which synchrotron SAXS has become accessible because of various advances in data analysis, detectors and synchrotron sources [66,67]. A significant feature of synchrotron SAXS is that it probes microstructure on length scales from approximately 2 to 100 nm [68].

Synchrotron SAXS is an important tool because it permits the use of time-resolved SAXS (Tr-SAXS) experiments due to the high brilliance of synchrotron undulator sources, which have flux typically between 10^12^ and 10^14^ photons per second. Tr-SAXS experiments allow investigation of kinetic pathways of self-assembly, vesicle formation, and drug loading processes, as there is a millisecond time resolution. Rapid mixing stopped-flow techniques combined with Tr-SAXS, with dead times as short as 2 ms, can trigger kinetic processes. As another option for the study of dynamic processes, the combination of microfluidics is used, where time resolution is translated to spatial resolution by controlling flow rates and channel volumes [69].

Anomalous SAXS (ASAXS) represents a unique but very powerful synchrotron scattering technique for structural characterization of charged soft matter and biological systems, using contrast variation by measuring around the X-ray absorption edge of an element of interest, where the scattering factors change significantly. Only one sample is needed compared to multiple deuteration conditions in SANS contrast variation [69].

Size exclusion chromatography coupled with synchrotron SAXS (SEC-SAXS) enables the selective observation of target molecules in multi-component systems by directly injecting the eluted solution from a SEC column into a SAXS cell, allowing measurement of just-separated components prior to their re-aggregation. SEC-SAXS was initially considered feasible only with synchrotron-based SAXS instruments due to the high beam intensity required for flow measurements, with the first system developed by David and Pérez at SOLEIL, after which the technique spread widely across synchrotron facilities worldwide [70].

#### 2.1.3. Small-Angle Neutron Scattering

SANS uses neutrons to investigate nanoscale structures through their interaction with atomic nuclei rather than electron clouds, as in SAXS [38,51]. Because it operates at small momentum transfer values, it is highly sensitive to nanoscale heterogeneities and variations in scattering length density between different phases or regions of a sample. This contrast, when measured, allows SANS to reveal information about particle morphology, pore structure, interfaces and internal organization in complex systems [71].

One of the most important advantages of SANS is its sensitivity to isotopic composition, particularly the large difference between hydrogen and deuterium scattering behavior [72]. This enables contrast variation techniques through isotopic substitution, commonly achieved using H_2_O/D_2_O mixtures or deuterated compounds, which can selectively enhance or suppress scattering from specific components. As a result, SANS is uniquely capable of distinguishing different regions within multicomponent systems and determining structural accessibility, pore connectivity or fluid penetration behavior [71]. In porous materials, contrast variation can be used to identify whether pores are open or closed and to investigate pore filling, adsorption processes and the effects of chemical treatments on internal structure.

USANS provides ensemble-averaged, nondestructive structural information on undiluted concentrated slurries and is sensitive to adsorption-saturation-dependent changes in particle aggregation and fractal organization, enabling indirect validation of adsorption-controlled dispersion states through their resulting microstructural signatures [73]. It is also used as a complementary structural technique to resolve nanoscale and submicron particle morphology and aggregation in mesoporous silica adsorbents, linking synthesis-dependent structural evolution to accessible surface and pore architecture and thereby supporting interpretation of adsorption performance [74]. In addition, when combined with rheology, USANS enables indirect determination of how binder adsorption regimes govern the evolution of particle agglomeration and polymer-mediated network formation in Si-based electrode slurries under flow [75]. Finally, in neutron scattering applications, USANS combined with SANS can distinguish methane-accessible from inaccessible pores via contrast matching, revealing how confinement drives pore-size-dependent adsorption, densification, and nano-cluster formation of methane within nanopores [76].

An important characteristic of SANS is that the technique detects only scattering contrast rather than direct images of objects, meaning that similar scattering patterns may correspond either to particles dispersed in a medium or to voids of comparable dimensions within a homogeneous matrix. Therefore, meaningful structural interpretation generally requires the use of appropriate physical and mathematical models during data analysis [77]. Compared with X-rays, neutron beams possess much lower energy, making SANS especially suitable for studying radiation-sensitive materials such as biomaterials, polymers, soft matter, and organic nanostructures without causing significant sample damage. Neutrons also penetrate deeply into matter, enabling the investigation of thick samples and the use of complex experimental environments that may be difficult to achieve with SAXS. This high penetration capability makes SANS valuable for studying hybrid organic–inorganic systems, hydrated materials, polymer particles and surface ligand conformations [51]. SANS experiments are typically performed using monochromatic neutron beams together with position-sensitive detectors that record scattering intensity profiles over a range of q-values. Measurements collected at multiple sample-to-detector distances are often combined in order to extend the accessible q-range and probe structures across several nanoscale length scales simultaneously [72,77].

Reliable SANS measurements require extensive correction and calibration procedures, including background subtraction, detector normalization, detector response correction, radial averaging and absolute intensity calibration. Following these corrections, the resulting scattering curves are analyzed using specialized modeling software to extract quantitative structural information such as particle size, pore dimensions, surface area and spatial organization. Another important advantage of SANS is its flexibility under controlled experimental conditions, allowing measurements during temperature variation, shear deformation, rheological testing or other dynamic environments. This capability enables in situ studies of structural evolution and nanoscale dynamics in complex materials and solutions [77].

#### 2.1.4. Porod’s Law and Fractal Interfaces

Porod’s law constitutes the general interpretation of small-angle scattering from porous solids during adsorption phenomena. For an ideal two-phase system with sharply defined, smooth interfaces, Porod provided a theoretical demonstration through his equation that the scattering originates solely from interfaces. When the interfaces are sharp relative to the length scale 1/q, the scattering profiles follow a power law in the limit q >> dx^−1^, where the prefactor is directly proportional to the specific surface area S_v_. Specifically, the differential scattering cross section per unit volume $\frac{d\Sigma \;}{V\;d\Omega }$ decays as q^−4^. Porod’s equation is the following [78]:

(8) $$limqdx>>1\;\frac{d\Sigma \;(q)\;}{V\;d\Omega }\;=2\pi (\Delta \rho )2\;Sv\;q^{-4}=\;Kq^{-4},\;qdx>>1$$

where *S_v_* is the specific surface area, $\frac{d\Sigma \;}{V\;d\Omega }$ is the differential scattering cross section per unit volume, *K* is the Porod constant and *Δρ* is the difference in scattering length density between the solid and pore phases.

If absolute intensity is unknown and the data extend to low enough *q*, it is possible to use the following Equation (9) in order to find the Porod invariant *Qp*:

(9) $$Q_{p}=\int_{0}^{\infty \;}q^{2}\;dq[\frac{d\Sigma \;\;}{V\;d\Omega }]=\;2\pi^{2}\;(\Delta \rho {)}^{2\;}\varphi_{s}\;(1-\varphi_{s})$$

where *φ_s_* is the volume fraction of the solid phase.

As a result of Equation (9), the specific surface area *S_v_* is possible to be calculated via Equation (10), which uses the relative scattered intensity *I*(q):

(10) $$S_{v}=\;\pi \;\varphi_{s}\;(1-\varphi_{s})\;limqdx>>1\;q^{4}\frac{I(q)}{Qp}$$

However, a critical refinement for real adsorbents emerged from the observation that many porous materials, such as lignite coals, exhibit scattering exponents with non-integer values between −3 and −4, rather than the classical −4 [79]. This deviation is not an artifact but a signature of surface fractality [80]. Bale and Schmidt demonstrated that for a pore boundary with a fractal (Hausdorff) dimension D (where 2 < D < 3), the asymptotic scattering follows I(q)∝ q ^−(6−D)^, generalizing Porod’s law. In this framework, a smooth Euclidean surface corresponds to D = 2 and the classical q^−4^ law. For a fractal surface, the specific surface area becomes length-scale dependent, and the fractal dimension can be directly obtained from the power-law slope. This fractal interpretation has been successfully applied to describe the rough internal surfaces of Vycor porous glass, where dry samples typically exhibit surface fractal dimensions around 2.5 ± 0.1 [32,81].

While deviations between −3 and −4 yield descriptive context regarding surface roughening, a highly significant alternative structural deviation occurs when the high-$q$ asymptotic power-law decay drops significantly faster than the classical Porod baseline, yielding an apparent slope larger than 4, for example, *I*(q)∝ *q*^−*α*^ where *α* > 4 is the development of a diffuse transition layer over a localized thickness across which the localized scattering length density varies continuously, rather than undergoing a mathematically sharp step-function jump [82,83,84].

In hybrid materials and modified porous matrices, such as organo-modified mesoporous molecular sieves (organo-MSU-X), the organic functional groups co-condensed or covalently grafted onto the pore walls possess an electron or scattering length density intermediate between that of the dense inorganic solid matrix and the empty pore voids [82,83]. This spatial distribution creates a continuous density gradient at the phase boundary that systematically dampens the scattering at high angles [84,85]. To quantitatively model this diffuse boundary layer, the continuous density transition along the normal to the interface (*z*-axis) is represented by a normalized profile function h(*z*) that varies from 0 to 1 across the thickness [84]. The total scattering intensity, *I*(*q*), is written as the product of the ideal sharp-interface scattering, *I_sharp_*(*q*) or *I_id_*(*q*), and a continuous exponential damping factor, *H*(*q*)^2^:

(11) $$I(q)=\;I_{sharp\;}(q){\left|H(q)\right|}^{2}\approx \;\frac{{2\pi (\Delta \rho )}^{2}\;Sv}{q^{4}}{\left|H(q)\right|}^{2}$$

The damping factor ${\left|H(q)\right|}^{2}$ is mathematically defined as the square of the Fourier transform of the derivative of the transition profile, *h*′(*z*):

(12) $${\left|H(q)\right|}^{2}\;=\left|\int_{-\infty }^{\infty }h^{\prime }\;(z)\;e^{-\;iqz}\;\right|{dz}^{2\;}$$

The specific mathematical expression for this damping factor depends directly on the geometric profile of the density gradient. If the gradient across the interface is modeled as a Gaussian function with standard deviation σ, the derivative is written as:

(13) $$h^{\prime }(z)\;=\;\frac{1}{\sqrt{2\pi \sigma }}\;exp\;(-\frac{z^{2}}{{2\sigma }^{2}})$$

resolving the damping factor to a Gaussian decay ${\left|H(q)\right|}^{2}=exp(-\sigma^{2}q^{2})$ and yielding the modified intensity expression:

(14) $$Ι(q)\approx \frac{2{\pi (\Delta \rho )}^{2}S}{q^{4}}exp(-\sigma^{2}q^{2})$$

Conversely, if the density varies linearly across a transition zone of defined thickness *t*, the damping factor resolves to:

(15) $${\left|H(q)\right|}^{2}\;={\left[\frac{sin(\frac{qt}{2})}{\left(\frac{qt}{2}\right)}\right]}^{2}$$

To experimentally determine the variance of the density gradient *σ*^2^ and extract the ideal Porod constant *Κ_p_*, Ruland proposed a linear approximation for the exponential damping factor when *q_σ_* is relatively small (*q_σ_* < 1) [84]. By taking the natural logarithm of Ruland’s modified Porod equation, the relationship is formulated as a linear relationship used to fit experimental data:

(16) $$ln[{I(q)q}^{4}]=ln(K_{p})-\;\sigma^{2}q^{2}$$

A plot of $ln[I(q)q^{4}]$ versus q^2^ (referred to as a modified Porod or Ruland plot) in the high-*q* regime yields a straight line with a distinct negative slope equal to *σ*^2^. In contrast, pristine control materials with sharp, smooth step-function boundaries adhere to classical Porod behavior and produce a flat profile with a slope of zero. From the extracted standard deviation, the physical thickness (*E*) of the interfacial transition layer can be precisely calculated based on the geometry of the density profile, where

(17) $$Ε=\sqrt{12}\;\sigma \approx 3.46\sigma$$

for a linear profile and

(18) $$Ε=\sqrt{2\pi }\;\sigma \approx 2.51\sigma$$

for a Gaussian profile [82,83,84].

When organic functional groups (such as methyl, ethyl, and mercaptopropyl groups) are successfully grafted onto pore surfaces, the calculated physical thickness (*E*) obtained via SAXS/SANS quantitatively matches the theoretical molecular length of the respective organic functional groups. This serves as non-destructive, physical proof that the modifier molecules are uniformly localized and covalently linked directly at the pore-matrix interface. Furthermore, this physical modeling directly confirms that the organic groups do not aggregate into isolated, bulk segregated organic phases within the pore centers or outside the mesoporous channels, validating structural orientations previously suggested by solid-state ^29^Si CP/MAS NMR and FT-IR spectroscopies.

Conversely, an entirely different structural regime is encountered when the power-law scattering exponent becomes shallower than 3 (*α* < 3), a region that cannot be explained by surface-roughening or diffuse-interface approximations. A power-law slope smaller than 3 signifies a volume (or mass) fractal structure. While surface fractals represent a rough, highly convoluted two-dimensional interface bounding a dense three-dimensional bulk (3 < *α* < 4), a volume fractal describes a highly porous, loosely aggregated network or poorly ordered system where the total mass (*M*) enclosed within a sphere of radius (*R*) scales anomalously with a non-integer mass fractal dimension *D_m_*, such that [84]:

(19) $$M(R)\propto R^{Dm}\;\mathrm{where}\;D^{m}\;<\;3$$

Under these conditions, the measured power-law intensity directly tracks the mass scaling configuration of the system, where the scattering power-law exponent is equivalent to the volume fractal dimension (*α* = *D_m_*). Highly porous polymer networks, swollen hydrogels, aerogels, or complex colloidal nanoaggregates frequently display these signatures during adsorption, gelation, or swelling processes. Systematically monitoring the transitions of the power-law slope across the distinct thresholds of *α* < 3 (volume fractals), 3 < *α* < 4 (surface fractals), *α* = 4 (sharp Euclidean boundaries), *α* > 4 (diffuse transition interfaces) allows for a unified structural understanding of network reorganization, cluster growth, and film accumulation pathways in real-time in situ studies.

The real power of Porod analysis in adsorption studies is realized through in situ contrast-matching experiments. By selecting an adsorbate (such as dibromomethane for SAXS or a H_2_O/D_2_O mixture for SANS) with a scattering length density equal to that of the solid matrix, the scattering from filled pores is suppressed, making empty pores the dominant scatterers. For nitrogen in mesoporous silica, the neutron scattering length densities of solid silica and liquid nitrogen are very similar (3.32 × 10^10^ vs. 3.25 × 10^10^ cm^−2^), which also allows for near-ideal contrast matching [85]. This technique permits the direct observation of pore filling and emptying as a function of relative pressure *p*/*p_o_*.

**Figure 2:**
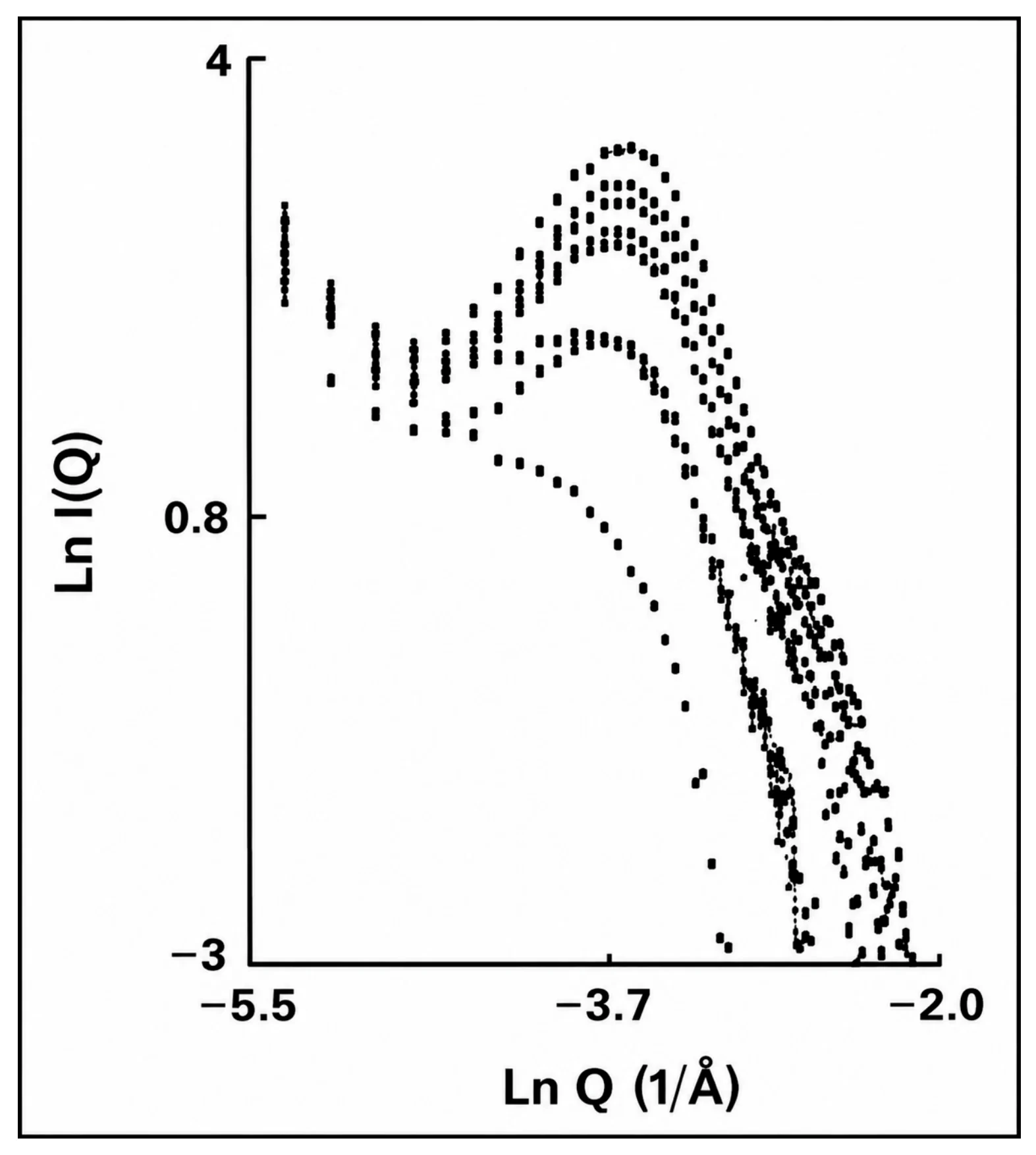
Porod plots for Vycor during dibromomethane adsorption at *p*/*p_o_* = 0 to 0.95 (top to bottom). Adapted from Mitropoulos et al. [32].

**Figure 3:**
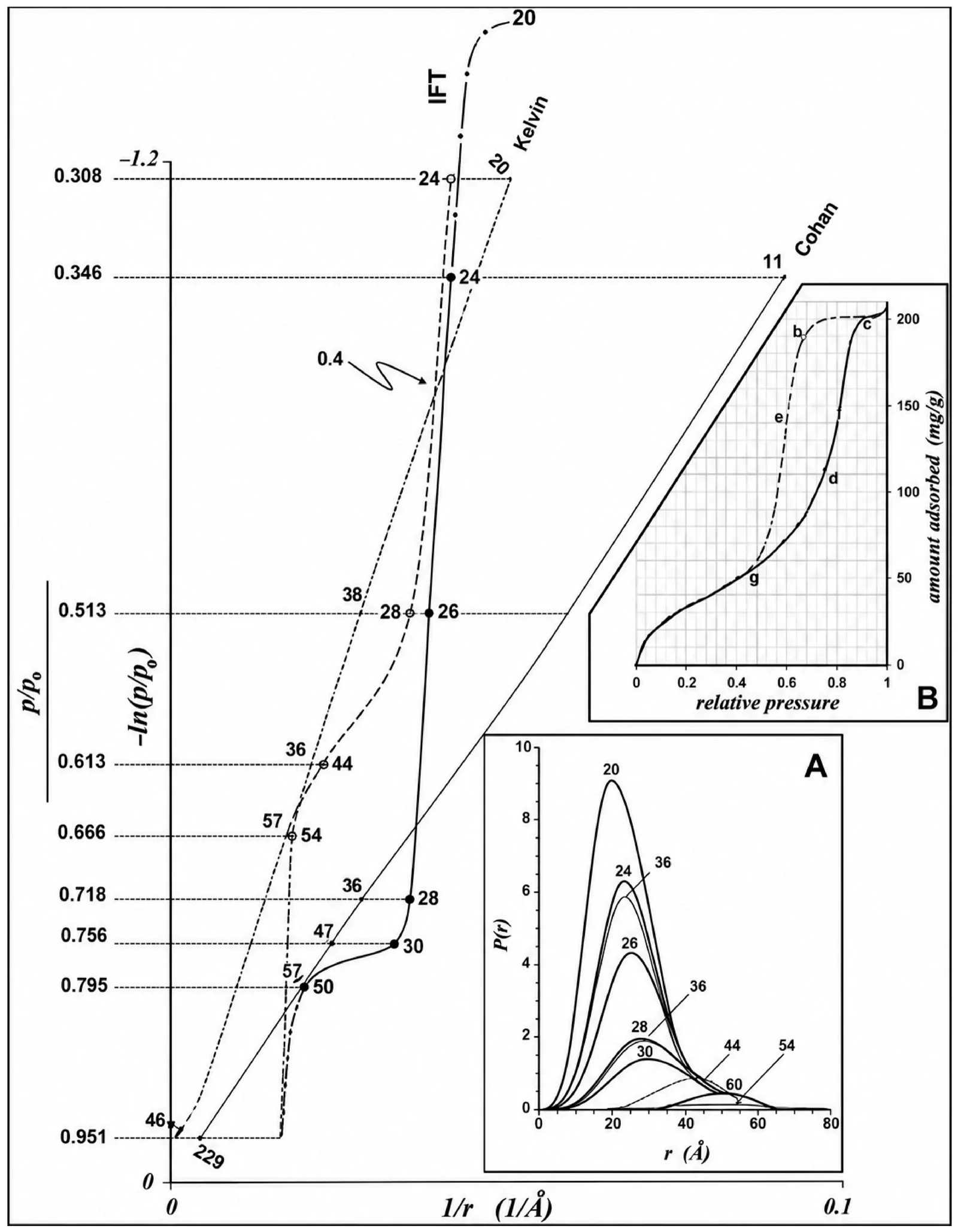
Comparative evaluation of capillary condensation and evaporation kinetics: classical Kelvin-Cohan (KC) model predictions versus the experimental Indirect Fourier Transformation (IFT) reconstruction. Solid and dashed curves denote the adsorption and desorption pathways, respectively, with corresponding calculated pore radii indicated at key relative pressures (p/p_0_). The thermodynamic IFT trajectory mirrors the macroscopic hysteresis, contrasting with the linear 2:1 relation dictated by the Kelvin and Cohan equations. (**Inset A**): Evolution of the pair distance distribution function P(r) as a function of CH_2_Br_2_ loading during adsorption (thick lines) and desorption (thin lines). (**Inset B**): Experimental adsorption–desorption isotherm of CH_2_Br_2_ on Vycor porous glass showing critical transition points (a; p^a^/p_0_ to c; p^e^/p_0_). Adapted with permission from Mitropoulos [86].

In general, during the procedure of adsorption, a multilayer film is formed. As a result, the initially rough pore surface becomes gradually smoother and a decrease in the surface fractal dimension is observed. This ‘defractalisation’ is caused by the Porod slope moving from values between −3 and −4 towards the Euclidean limit of −4. For example, for Vycor, defractalisation occurs at an adsorbed film thickness of approximately 15 Å, corresponding to the upper cutoff length scale of the surface roughness (Figure 2). At higher relative pressures, when capillary condensation begins, a sharp drop in scattered intensity from the peak region is observed, allowing the Kelvin radius of the pore class being filled to be determined. This has enabled the reconstruction of entire adsorption isotherms directly from SAXS data by plotting either the intensity at the correlation peak *I*(*Q_p_*) or the Porod slope versus *p*/*p_o_* (Figure 3) [32].

Perhaps the most striking insight from in situ scattering concerns the desorption process. Using contrast-matched SANS, Li et al. [81] showed that during desorption, a new low-q power-law component appears, with a fractal dimension of 1.75 ± 0.1 for Vycor, 1.85 ± 0.1 for Ludox, and 2.0 ± 0.1 for a silica sol–gel. This scattering arises from ramified, fractal-like clusters of empty pores connected to the external surface, providing direct diffraction evidence for a percolation-controlled drainage mechanism. In contrast, during adsorption, such fractal features are absent, consistent with a process of random, isolated pore condensation. Similar power-law scattering from vapor-filled void clusters (exponent 2.27 ± 0.05) has been observed during nitrogen desorption from controlled-pore glass CPG-10-75, further supporting the percolation hypothesis [85].

Importantly, when Porod’s law is not strictly followed (such as slopes not equal to −4), quantitative information can still be extracted. For fractally rough surfaces, the Porod constant can be evaluated at the upper limit of the fractal regime to approximate mean chord lengths, or alternatively, a smoothing function can be introduced to account for diffuse interfaces when slopes become steeper than −4 [32,78]. Collectively, these developments transform Porod analysis from a simple tool for surface area measurement into a powerful method for probing the dynamic, length-scale-dependent geometry of pore interfaces during adsorption and condensation.

The structural implications of the scattering exponent a are summarized in Table 1.

#### 2.1.5. Guinier’s Law and the Radius of Gyration

As described in the previous subsection, Porod’s law describes the high-*q* asymptote of the scattering curve. Nevertheless, information about the overall size of scattering objects is contained in the low-*q* region. Guinier’s approximation mentions that for *q*R_g_ << 1, the scattered intensity *I*(*q*) from a dilute system of monodisperse particles follows the following equation [40]:

(20) $$I(q)=I(0)\;exp\;(-\frac{q^{2}{\;R_{g}}^{2}}{3})$$

where $R_{g}$ is the radius of gyration and *I*(0) is the forward scattering intensity, proportional to the square of the contrast and the volume fraction of scatterers. A Guinier plot (ln *I*(*q*) vs. *q*^2^) yields a straight line with slope $-\frac{{R_{g}}^{2}}{3}$, providing a model-independent estimate of particle or pore dimensions [41].

**Figure 4:**
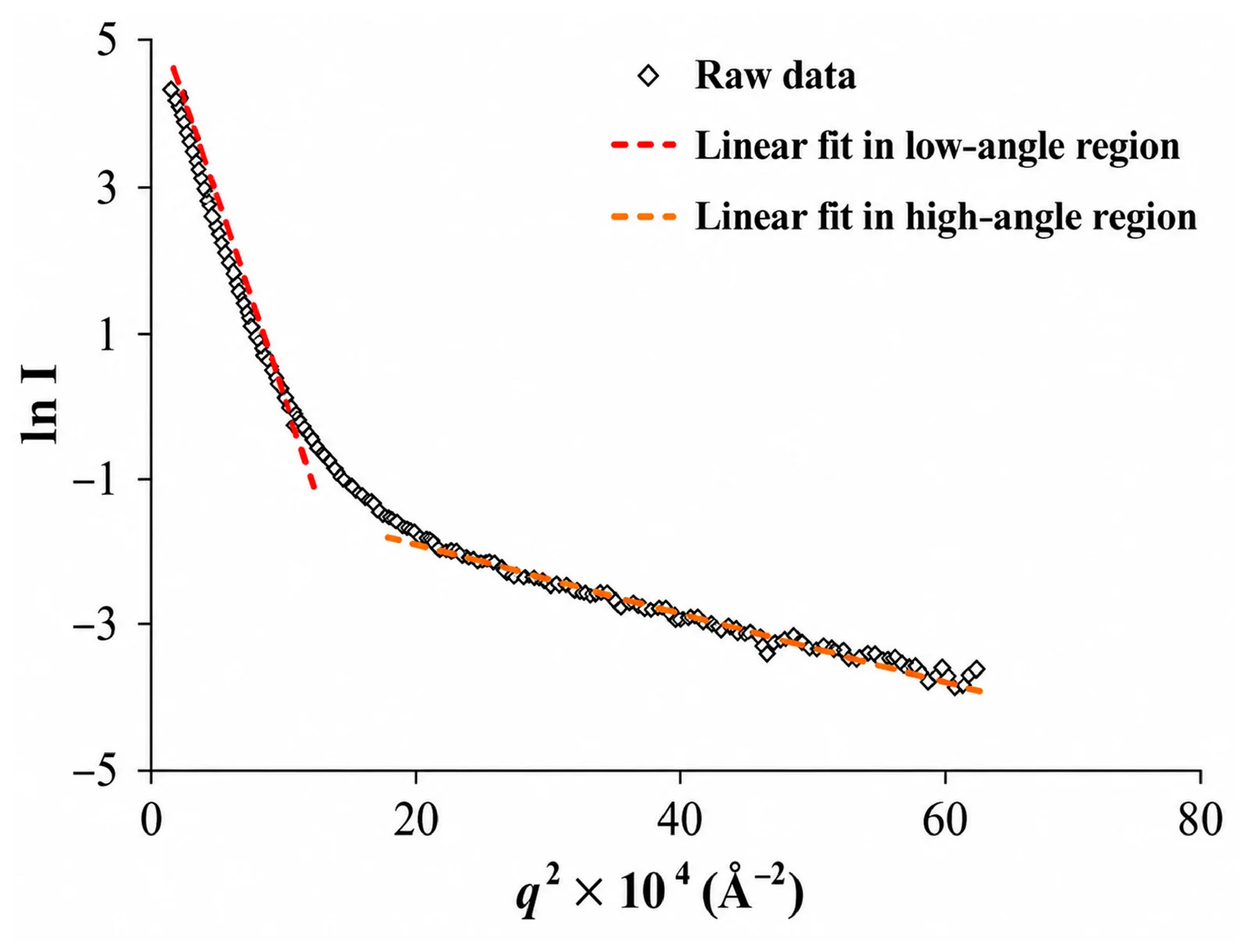
An example of the Guinier plot of SAXS data. Dashed lines indicate the separate fits of the Guinier equation to the low- and high-angle regions of the raw data, allowing the extraction of two distinct radius of gyration values [88].

For porous materials $R_{g}$ can be interpreted as the average pore radius or the correlation length of the pore-solid interface, depending on the scattering contrast [49]. Guinier’s approximation is accurate only in the occasion of ${q\;R}_{g}\leq \;1.3.$ Deviations from linearity in the Guinier plot indicate polydispersity, aggregation, or interparticle interactions (Figure 4). Guinier’s law is also a prerequisite for calculating the Porod invariant, as it allows extrapolation of the scattering intensity to *q* = 0 when experimental data do not reach sufficiently low *q* [31,41].

Together with Porod’s law, Guinier’s law provides a complementary framework: the low-*q* regime yields size parameters, while the high-*q* regime yields surface and interface characteristics.

### 2.2. Contrast Variation and Contrast Matching

While the previous section of this review described in detail the fundamentals of both SAXS and SANS for homogeneous systems, real biological and materials science samples are invariably multi-component complexes. To resolve the individual contributions of each component, such as for instance protein and nucleic acid, or catalyst and support, one must exploit the fact that the scattering intensity depends on the square of the difference in scattering length density (SLD) between solute and solvent. This is the principle of contrast variation. Contrast variation exploits the fact that the scattering intensity *I*(q) depends on the square of the difference in SLD between the solute and the solvent Δρ^2^. When the SLD of a molecule equals that of the solvent (Δρ = 0), its coherent scattering intensity becomes zero at that specific contrast match point [89]. In small-angle neutron scattering (SANS), this approach is particularly powerful because the neutron scattering length varies randomly between isotopes and can be positive or negative, unlike X-rays. Specifically, replacing ^1^H and ^2^H (deuterium) changes the SLD without significantly altering the molecule’s physical properties, while the solvent SLD can be continuously tuned by mixing H_2_O and D_2_O [89,90]. This allows the experimenter to adjust the buffer SLD to exactly match one component in a complex, a condition called contrast matching, so that by measuring a series of samples with increasing D_2_O content (a contrast variation series), each partner can be highlighted in turn while the others become invisible [89].

In SAXS, contrast variation originated using glycerol to change solvent electron density, but it became far more powerful in SANS because H_2_O/D_2_O mixtures provide a wider tunable range covering all major biological macromolecules [91]. In SAXS, however, one can add inert contrast agents such as sucrose (which is highly soluble, inert, and does not absorb X-rays strongly) to increase the solvent electron density [92]. For instance, in a protein-nucleic acid complex, when the solvent density matches that of the protein at ~50–65% *w*/*v* sucrose, the protein becomes invisible and only the nucleic acid contributes to the scattering. A critical practical distinction is that protein–nucleic acid complexes (e.g., protein-DNA, protein-RNA) do not require deuteration in SANS either because the protein matches at ~40% D_2_O and DNA/RNA at ~65% D_2_O, which are sufficiently different. Similarly, protein–detergent complexes typically need no deuteration because detergents are rich with CH_2_-. However, for protein-protein complexes, deuteration of one component is necessary-random deuteration of ~60% of non-exchangeable hydrogens shifts its match point to ~88% D_2_O, creating sufficient contrast between the two proteins [89].

From a full contrast series (typically five different D_2_O or sucrose concentrations), the scattering of a multi-component complex can be decomposed into three terms: the scattering from component A alone, from component B alone, and a cross-term containing their relative positions [90,92]. For a two-component particle, the contrast dependence of the radius of gyration follows the Stuhrmann equation [91]:

(21) $${R^{2}}_{g}=\;{R^{2}}_{c}+\;[\frac{\alpha }{\Delta \rho }]\;-\;[\frac{\beta }{\Delta \rho^{2}}]$$

where *α*: is positive when the low-density component is in the center and negative when the high-density component is in the center, while *β*: is non-zero when the centers of mass of the components do not coincide (dipolar structure). *R_c_* is the radius of gyration of the particle’s overall shape at infinite contrast and *Δρ* is the difference in SLD between the solute and the solvent.

When full data decomposition is not possible, for example due to limited contrast range or aggregation, the Stuhrmann plot and the parallel axis theorem still yield the radius of gyration of each component and their center-of-mass separation [90]. Programs such as MONSA or MulCh can then reconstruct the separate shapes of the components and their spatial arrangement [92].

A classic example is the *E. coli* ribosome large subunit, which showed RNA in the center and proteins on the surface. Cauliflower mosaic virus (three contrasts) revealed protein and DNA on concentric outer shells [91]. Bacteriophage MS2 capsid and RNA were reconstructed and validated against cryo-EM. In protein-nucleic acid SAXS, CV determined stoichiometry, RNA bending, and salt-induced DNA unwrapping from nucleosomes in real time [92]. Time-resolved SANS followed GFP degradation by the PAN-20S proteasome, amyloid fibril elongation, and subunit exchange in αβ-crystallin. For membrane proteins, deuterated detergents enabled studies of GluA2 and the bacterial holo-translocon. For lipid nanoparticles, global fitting at five contrasts located an anti-inflammatory compound in the shell of MC3 LNPs. For non-spherical nanoparticles, the Novavax RSV vaccine was shown to consist of a cylindrical micellar core with five protein trimers [89]. In materials science, CV-SAXS with tetrabromoethane/DMSO matched the carbon support electron density, revealing true Pt nanoparticle size (2.6–2.7 nm) even at low loadings where carbon pore scattering would otherwise obscure it [93].

Triple isotopic substitution measures distances between labeled components via the cross-term 2A1A2, and triangulation of 105 distances mapped all proteins in the small ribosomal subunit. Nuclear spin contrast variation uses a polarized neutron beam and dynamic nuclear polarization to vary contrast without changing solvent or labeling, locating two tRNAs on the E. coli ribosome at low occupancy [91].

Labile hydrogens (O–H, N–H, S–H) exchange with solvent, changing the biomolecule’s SLD with D_2_O content [90]. The match point is found by plotting √*I*(0) vs. % D_2_O [89,90]. For Bio-SANS, each component should be ≥10 kDa, requiring ≥200 µL of 2 mg/mL. Neutrons cause no radiation damage, so the same sample can be re-used for SAXS as a control [90]. SAXS is recommended to check for deuterium-induced structural changes, as X-rays cannot distinguish H from D. Challenges include deuterium-induced aggregation [89,91]. Finally, in vivo contrast variation in Bacillus subtilis made only the cell membrane visible by growing cells in deuterated media with re-introduced hydrogenated fatty acids [89]. Contrast variation remains the main reason scientists choose SANS over SAXS to determine the relative positions of partners within a hetero-oligomer [90].

Contrast matching is a special case of contrast variation, a technique particularly powerful in SANS. In SANS, solution conditions are adjusted so that the scattering-length density (SLD) of one component matches that of the solvent, rendering it practically “invisible” and allowing the scattered signal to be dominated by the other component(s) of a complex [94,95]. The fundamental quantity governing this effect is the contrast *Δρ* = ρ_molecule_ − ρ_solvent_. For neutrons, contrast manipulation is exceptionally powerful because neutrons are scattered by atomic nuclei, and different isotopes, notably ^1^H (protium) and ^2^H (deuterium), have very different scattering lengths: deuterium has a large positive value, while hydrogen has a negative value about half that magnitude. By varying the H_2_O/D_2_O ratio in the solvent, it is possible to change both ρmolecule and ρsolvent without altering chemical composition. The match point is the specific D_2_O fraction where *Δρ* = 0 [96,97].

The same principle can be applied to SAXS, though contrast here is determined by electron density rather than nuclear scattering lengths. A concentrated sucrose solution (65% *w*/*v*) has an electron density closely matching that of the average protein. When a protein is placed in such a solvent, its overall scattering is suppressed, but any heavy atoms bound to the protein (such as Pb^2+^) remain visible due to their much higher electron density. The resulting scattering curve is then dominated by the Debye formula for a set of point scatterers, allowing direct extraction of interatomic distances without a full atomic model [94].

For a two-component biomolecular complex (for example, protein–protein or protein–nucleic acid), SANS contrast variation data can be decomposed into core scattering functions: *I*11(q) (component 1 alone), *I*22(q) (component 2 alone), and *I*12(q) (the cross-term encoding their relative arrangement). These are obtained via multiple linear regression from multiple contrast points. At a component’s match point, the cross-term and the other component’s scattering vanish, leaving only the visible component’s scattering [50]. The forward scattering *I*(0) is proportional to Δρ^2^ for homogeneous molecules; a plot of √[*I*(0)/N] vs. fD_2_O gives a V-shaped function intersecting zero at the match point. Analysis of *I*(0) across contrasts helps identify sample dissociation or aggregation induced by D_2_O. The radius of gyration Rg, for a two-component complex, varies with contrast, and this dependence is analyzed using a Stuhrmann plot (Rg^2^ vs. 1/Δρ), yielding parameters such as Rc (infinite-contrast Rg) and the distance between centers of mass via the parallel axis theorem.

Contrast matching is also widely used to study porosity. In coal, filling open pores with an H_2_O/D_2_O mixture matching the coal’s SLD eliminates scattering from open pores; residual scattering then originates from closed pores or internal density fluctuations. For Pittsburgh No. 8 coal, after contrast matching, the scattering dropped more than tenfold (Porod invariant from 1.8 × 10^−3^ to 1.1 × 10^−4^), demonstrating the absence of a connected pore system [96]. Similarly, in fluid catalytic cracking (FCC) equilibrium catalysts contaminated with coke, contrast matching with deuterated methanol (ρ~5.8 × 10^14^ m^−2^) revealed that much soluble coke is trapped in closed pores. Fresh catalyst showed negligible closed porosity, whereas coked catalysts exhibited significant closed porosity. A small coke content (≥2 wt%) caused most pore blockage, suggesting pore-mouth occlusion [97]. For X-ray-based pore studies, halogenated organic liquids (such as bromoform, dibromomethane) can match the electron density of silica or carbon. Varying adsorbate loading or surface modification (silylation) yields information on pore morphology, surface roughness, and pore size. For example, silylation of fumed silica changed the Porod slope from fractal (α~3.45) to smooth (α = 4), providing surface smoothing (“defractalization”) [87].

Contrast-matching SANS has been used to reveal molecular packing in self-assembled fibers of low molecular weight gelators (LMWGs). By comparing scattering from differently deuterated analogs (for example, deuterated naphthalene vs. deuterated terminal phenylalanine) in D_2_O, the contribution of each segment to the scattering curve can be identified. In the case of 2NapFF, at high pH the system formed hollow cylinders (wall 2.1 nm, core radius 1.65 nm), but upon lowering pH to form a gel, the hollow core collapsed and fibers became elliptical. The lack of sharp contrast effects indicated that molecular packing in these gels is not well-ordered, explaining why predictive design rules for LMWGs remain elusive [95].

The single largest barrier to successful SANS contrast variation is the need for a consistent set of monodisperse samples, as dissociation, aggregation, or flexibility induced by D_2_O can compromise data. Including *I*(0) plots, Stuhrmann plots, and composite scattering functions in publications is strongly recommended to demonstrate sample homogeneity and data reliability before proceeding to 3D modeling. In the X-ray contrast-matching approach using sucrose and heavy atoms, the method avoids the lower signal-to-noise of SANS and the complications of radioactive isotopes, and it does not require decomposing multiple datasets into separate scattering functions. It can be extended to any protein by engineering surface cysteines and attaching EDTA-metal moieties via disulfide bonds [94]. Contrast-matched SAXS with halogenated liquids or matched silylation agents is a practical tool for pore structure analysis accessible to labs without neutron facilities.

## 3. Fundamentals of Adsorption Phenomena

### 3.1. Classification of Adsorption Processes

#### 3.1.1. Physisorption and Chemisorption

Adsorption is a method used universally for the removal of different types of pollutants from industrial waste [98]. It is a surface phenomenon, opposite to absorption which is a bulk phenomenon [99], that takes place at an interface and it depends on interactions between the adsorbent and the adsorbate material [100,101,102] and it involves a gas or liquid phase [101], that is placed on the pores of a solid surface [103]. Adsorption can be classified into two categories: physisorption (physical adsorption) and chemisorption (chemical adsorption). During physisorption, weak interactions occur through physical forces, while during chemisorption, chemical bonding leads to stronger interactions and requires a higher enthalpy, about 10 times larger than that of physisorption [100,101]. Moreover, physisorption is often reversible, in contrast to chemisorption, which is often irreversible [100,103]. Physisorption and chemisorption are able to take place simultaneously [101]. Adsorption in general is affected by the temperature and surface area [104].

More specifically, physisorption is a surface phenomenon which is driven by weak intermolecular forces, mostly Van der Waals forces and is observed at low temperatures. The covalent interactions are non-existent, and no chemical reactions occur; therefore, the molecules maintain their identity. This means that physisorption is something that can be reversible under certain conditions. Physisorption can often be related to the way condensation occurs [105,106]. The molecules can form mono- or multi-layers or even fill completely micropores on the surface of the adsorbent, thus leading to the adsorbed phase being denser than the bulk phase that exists in the system [107]. Physisorption increases when pressure is increased and ends when the system reaches the condensation point. This type of adsorption is commonly analyzed using adsorption isotherms, which provide important information about surface area, pore size distribution, and pore volume of materials. The Brunauer–Emmett–Teller (BET) model is widely used to describe multilayer physisorption [108,109].

Chemisorption is a result of much stronger interactions, since there are chemical bonds; that is why it is often an irreversible process [109,110,111,112]. During chemisorption, the adsorbate forms a single layer on the adsorbent’s surface [109,110]. This type of adsorption is studied through the Langmuir model [109]. While physisorption is able to happen without the need for heating [112], chemisorption often requires activation energy. Chemisorption is temperature dependent and increases when temperature does [109]. It can occur through physisorption, since under the right conditions a molecule that is attached weakly to a surface can create a strong chemical bond. The surface area is a critical factor for the successful adsorption during chemisorption, as it contains a specific number of active sites, on which the chemical bonds are formed. Chemisorption comes to an end when all active sites are occupied [108,110,111].

The information above is summarized in Table 2 below:

#### 3.1.2. Gas vs. Liquid-Phase Adsorption

Gas phase adsorption is a separation process in which a gas component is removed from a gas stream by being attached to a solid surface [114,115]. Gas phase adsorption depends on the total gas composition and the partial pressure of each of its components [115,116]. Also, some gases can work competitively against the one that is supposed to be adsorbed and block some activated sites. Temperature plays a critical role in gas adsorption, since it affects the strength of the attachment between molecules and the surface [115,117]. A gas can be captured differently depending on whether there is a physisorption or chemisorption process going on. Gas adsorption is a technique widely used for the characterization of a porous material, mostly through the BET method, using nitrogen adsorption at 77 K [114]. Physisorption is mainly preferred so that the material will not undergo any changes in its molecular composition [117]. In microporous materials, adsorption does not strictly follow layer-by-layer growth but is often dominated by a pore-filling mechanism at low relative pressures, which is commonly described in gas physisorption systems using BET-derived approaches for surface area estimation [118]. Adsorption isotherms offer various structural information, such as the pore volume, the pore size distribution and the apparent internal surface area of the absorbent [119].

Liquid phase adsorption is a process in which dissolved components in a liquid (water or fuel) are removed through their accumulation on the surface of a solid adsorbent [120,121]. It is a process mainly used for removing impurities from liquids [122]. Adsorption in liquid systems is affected by multiple factors, some of them being interactions between the solvent and the dissolved species, competition among different solutes for available adsorption sites and the chemical nature of the adsorbent surface, particularly its functional groups [123]. In liquid phase adsorption, the equilibrium point is established more slowly, compared to gas phase adsorption, as it is mainly controlled by mass transfer through the liquid boundary layer within the pores of the adsorbent [120]. The liquid phase adsorption often uses functionalised or modified adsorbents, thus making it highly chemistry dependent (chemisorption) [123]. Because of this, liquid phase adsorption requires high selectivity [122].

The information above is summarized in Table 3 below:

### 3.2. Adsorption Mechanisms

Adsorption mechanisms involve a series of steps. Initially, adsorbate molecules are bound to active sites on the adsorbent’s surface, creating surface intermediates. These intermediates may then interact with other adsorbed substances, leading to reactions and formation of products, which end up desorbing from the surface. This mechanism, also known as the Langmuir-Hinshelwood mechanism, depends on factors such as surface coverage, competition between species and active sites, and whether the adsorption is reversible or irreversible [98,99]. Variations in surface composition and structural heterogeneity, arising from different synthesis routes and dopant incorporation, can significantly influence adsorption behavior by modifying surface reactivity and pore structure [100].

#### 3.2.1. Monolayer and Multilayer Adsorption

Monolayer adsorption generally occurs within a relative pressure range of 0.05–0.2. It is a fundamental concept in adsorption theory, whose capacity is calculated from the isotherm in order to estimate surface area, with the BET constant reflecting the strength of adsorbate-surface interactions [101,102]. This analysis provides a quantitative limit beyond which additional adsorption forms multiple adsorbate layers [103]. Energetically, monolayer formation is characterized by higher adsorption energies, which decrease noticeably once the surface is fully covered [104]. The maximum monolayer capacity is not constant but depends on factors such as ionic strength, particle size, and intermolecular interactions [105]. However, in practice, it is not clearly defined and depends heavily on the analytical method and assumptions used [102].

Multilayer adsorption can be described as the successive stacking of molecular layers on the adsorbent surface, where the total thickness of the adsorbed film increases along with the number of layers [106,113]. It is considered to begin gradually once the monolayer coverage is completed. The transition from monolayer to multilayer adsorption is continuous rather than sharply defined and usually occurs within the same relative pressure range as the monolayer adsorption [107,113].

#### 3.2.2. Pore Filling and Capillary Condensation

During adsorption, pore filling can occur, mostly during physisorption. The phenomenon is strongly influenced by pore structure, pore size, geometry and connectivity [109,110]. Generally, narrow micropores are filled first, while larger micropores and mesopores are filled as the adsorbate’s concentration increases, stating that pore filling is a concentration-dependent process [111,112]. Surface functional groups can both enhance adsorption through interactions, such as π-π electron donor-acceptor interactions, hydrogen bonding, electrostatic attraction and Lewis acid-base interactions, and limit it by forming water clusters that obstruct pore entrances [124]. Micropore filling can proceed via different mechanisms depending on the nature of the adsorbate–adsorbent interactions [115]. For nonpolar molecules, the adsorption field extends throughout the entire pore space, leading to a continuous filling process where adsorption increases gradually with pressure. In contrast, for polar molecules like water, the interaction with the pore walls may be weak, and adsorption occurs only after the formation of liquid-like clusters stabilized by intermolecular forces such as hydrogen bonding [112,115].

Adsorption in mesopores can be described through capillary condensation, a process where a vapor condenses into a liquid inside small pores at pressures lower than the bulk saturation pressure. Capillary condensation can be described using the Kelvin equation [114]:

(22) $$ln(\frac{p}{p_{s}})=-\frac{2\gamma V_{m}}{rRT}$$

where *γ* is the surface tension of the liquid, *V_m_* is the molar volume of the liquid, *r* is the radius of curvature, *R* is the universal gas constant and *T* is the absolute temperature in Kelvin.

**Figure 5:**
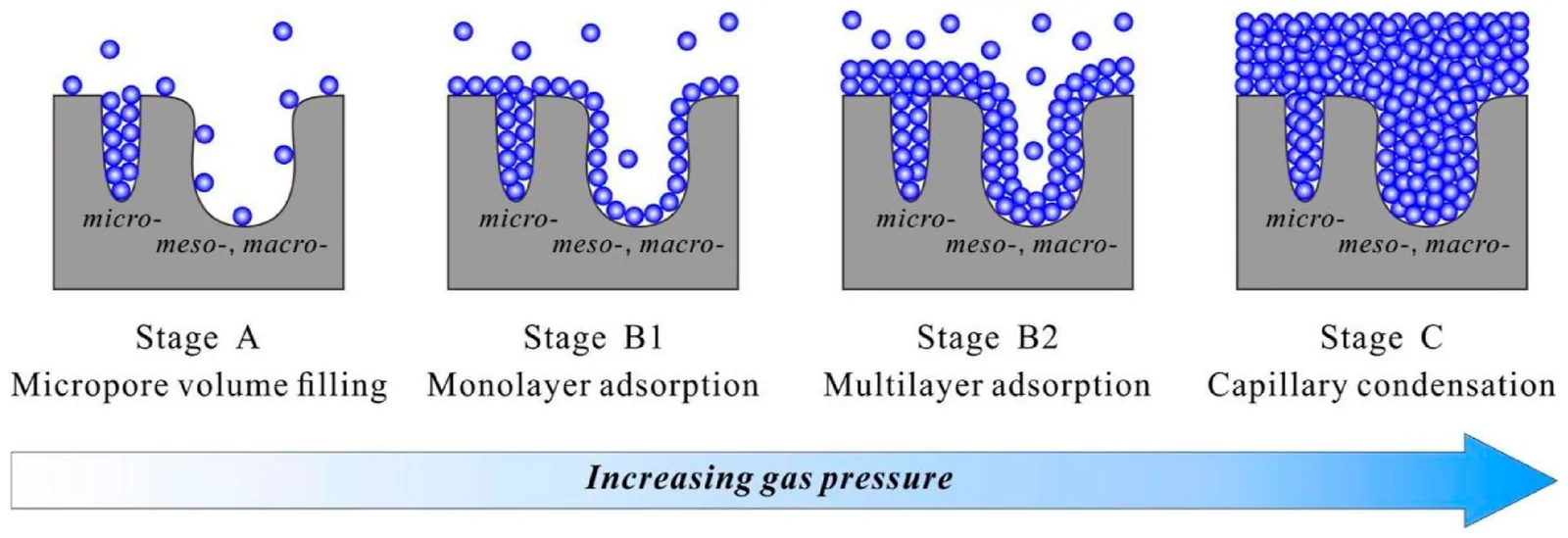
A schematic illustrating how micropores fill with adsorbate: the pores become occupied by the adsorbate prior to any significant coverage of the external surfaces. This pore-filling mechanism complements BET theory for gas physisorption in porous solids [125].

It relates the equilibrium vapor pressure in a pore to the curvature of the liquid-vapor interface, across which a pressure difference develops due to surface tension, leading to a reduction in the chemical potential of the confined liquid phase. This is what results in capillary condensation, and it is a mechanism that depends strongly on pore geometry [107,114]. In Figure 5, a schematic representation of pore filling is shown.

The occurrence of capillary condensation significantly enhances adsorption capacity but may also influence transport properties, as pore filling can either facilitate diffusion through multilayer formation or hinder flow due to pore blockage [118,119].

## 4. In Situ and Operando Small-Angle Scattering

In situ scattering techniques refer to structural characterization performed under controlled external stimuli, enabling direct observation of nanoscale evolution during processes such as phase transitions, self-assembly or deformation [120,121,122,123,126]. Operando methodologies extend this concept by correlating structural evolution with functional performance such as rheology, transport or reaction kinetics in real time [127,128,129,130,131]. Together, these approaches provide quantitative access to kinetic pathways involving nucleation, growth, aggregation and phase separation across soft, hard and biological matter systems [132,133,134,135]. In soft matter systems, in situ scattering captures gelation, micellisation and hierarchical self-assembly processes with time-resolved precision [136,137,138,139,140]. In complex fluids and composites, it is further revealed that deformation-induced anisotropy and structural rearrangements under mechanical or thermal stimulation directly link nanoscale evolution to macroscopic response [141,142,143].

Gas-phase in situ scattering experiments are conducted using sealed environmental cells that allow precise control of pressure, temperature and gas composition, enabling real-time monitoring of structural evolution in porous materials [144,145,146]. These setups are widely used to investigate adsorption-driven pore filling, density redistribution and hierarchical structural changes in nanoporous solids under operating conditions [147,148]. They are particularly effective for resolving nanopore evolution, gas uptake kinetics and thermally induced phase transformations in carbonaceous and oxide frameworks [149]. In addition, gas phase environments allow observation of precipitation, sintering and reaction-driven nanostructural evolution in solid-state systems under thermal treatment [150,151]. Overall, such experiments provide multiscale insight into porosity evolution and structural dynamics inaccessible by ex situ methods [152,153].

Liquid phase in situ SAXS and SANS experiments provide a versatile platform for probing colloids, polymers, biomaterials and liquid crystals under realistic processing conditions [154,155]. These measurements enable direct tracking of aggregation, gelation, phase separation and self-assembly pathways in complex fluids with high temporal resolution [156,157]. In polymeric and hydrogel systems, they capture network formation, fractal aggregation and solvent-driven swelling processes governing mechanical and structural properties [158,159,160]. In liquid crystalline systems, nanoparticle incorporation can induce multidomain nematic ordering and gelation through hierarchical fibrillar network formation [161,162,163,164]. Moreover, liquid phase scattering is essential for resolving structural organization in protein, lipid assemblies and biologically relevant soft matter systems under native conditions [165,166,167].

Time-resolved SAXS and SANS enable direct observation of kinetic pathways including nucleation, growth, phase separation and structural relaxation across timescales from milliseconds to hours [168]. In metallic and alloy systems, these methods quantify precipitation kinetics, coarsening behavior and phase decomposition during thermal or mechanical processing [169]. In soft matter, time-resolved scattering captures micelisation, gelation and hierarchical self-assembly processes governing macroscopic material properties [170,171]. Under mechanical deformation, it further reveals structural evolution such as lamellar reorganization and cavitation, directly linking nanoscale changes to mechanical response [172,173,174]. These techniques therefore provide a unified framework for connecting nanoscale dynamics with macroscopic rheology and mechanics [125,159].

Contrast variation in SANS enables selective visualization of individual components in multicomponent systems through isotopic substitution, most commonly hydrogen/deuterium exchange [175,176]. This approach allows separation of scattering contributions from polymers, solvents, proteins and nanoparticles within structurally complex materials [136,155,165,167]. Contrast matching is particularly powerful for resolving confined or interfacial structures, such as proteins in porous hosts or polymer coatings on nanoparticle surfaces [177]. It further enables isolation of bound polymer layers and internal interfaces in hierarchical composite systems, which are otherwise inaccessible by SAXS alone [178]. Overall, isotopic labeling provides a unique route to uncover hidden nanoscale architectures in complex soft and hybrid materials [157,165,175].

## 5. In Situ/Operando SAXS and SANS for Adsorption Studies

### 5.1. Soft Matter and Polymer Systems

Small-angle scattering techniques, especially when applied under in situ and operando conditions, allow the detection of structural rearrangements caused by adsorption in soft polymer assemblies, such as shell thickness, aggregation number and particle morphology [179]. Generally, SAXS and SANS provide powerful insight into polymer adsorption by tracking nanoscale structural changes such as interparticle correlations, structure factors and hierarchical ordering [180,181,182]. Through these techniques, conclusions on adsorption phenomena are drawn from structural evolution [180,182].

In polymer-soft systems, adsorption is connected to structural reorganization, where adsorption causes unfolding, extension or rearrangement, leading to interfacial packing, surface coverage and the coexistence of bulk and interfacial populations [183]. SAXS and SANS can capture how polymer or surfactant systems reorganize in a bulk solution, which indirectly determines their availability for interfacial adsorption [184].

**Figure 6:**
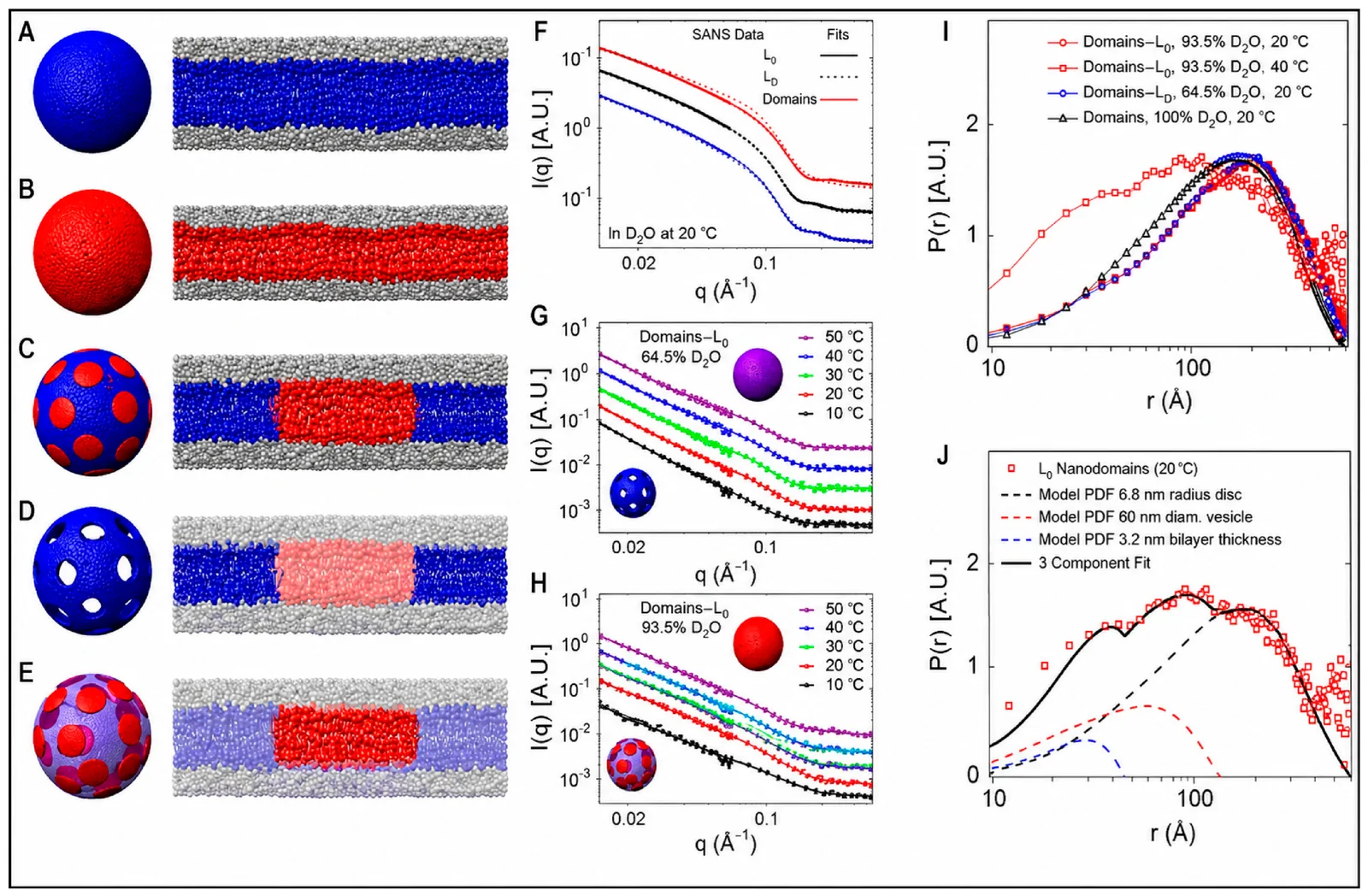
SANS measurements and neutron contrast-matching strategies used in this study. Transparent regions indicate parts of the bilayer that are contrast matched to the solvent and therefore effectively “invisible” to neutrons. (**A**) L_O_, (**B**) L_D_, (**C**) nanodomain-containing composition without contrast matching (“Domains”), (**D**) domain-containing composition contrast matched to highlight the LO_OO matrix (“Domains–L_O_”), and (**E**) domain-containing composition contrast matched to emphasize LD_DD nanodomains (“Domains–L_D_”). Blue denotes the L_O_ phase, while red denotes the LD_DD phase. (**F**) SANS data and corresponding fits for samples (**A**–**C**). (**G**) Temperature-dependent SANS data for sample D. (**H**) Temperature-dependent SANS data for sample E. (**I**) Pair distribution functions (PDFs) derived from SANS data, highlighting structural changes in the Domains–L_D_ sample above and below the miscibility temperature of 40 °C. (**J**) PDF of the Domains–L_D_ sample at 20 °C compared with a model PDF representing nanodomains approximately 3.2 nm thick and 6.8 nm in radius, distributed within a 60 nm diameter sphere. Error bars represent ±2σ [188].

**Figure 7:**
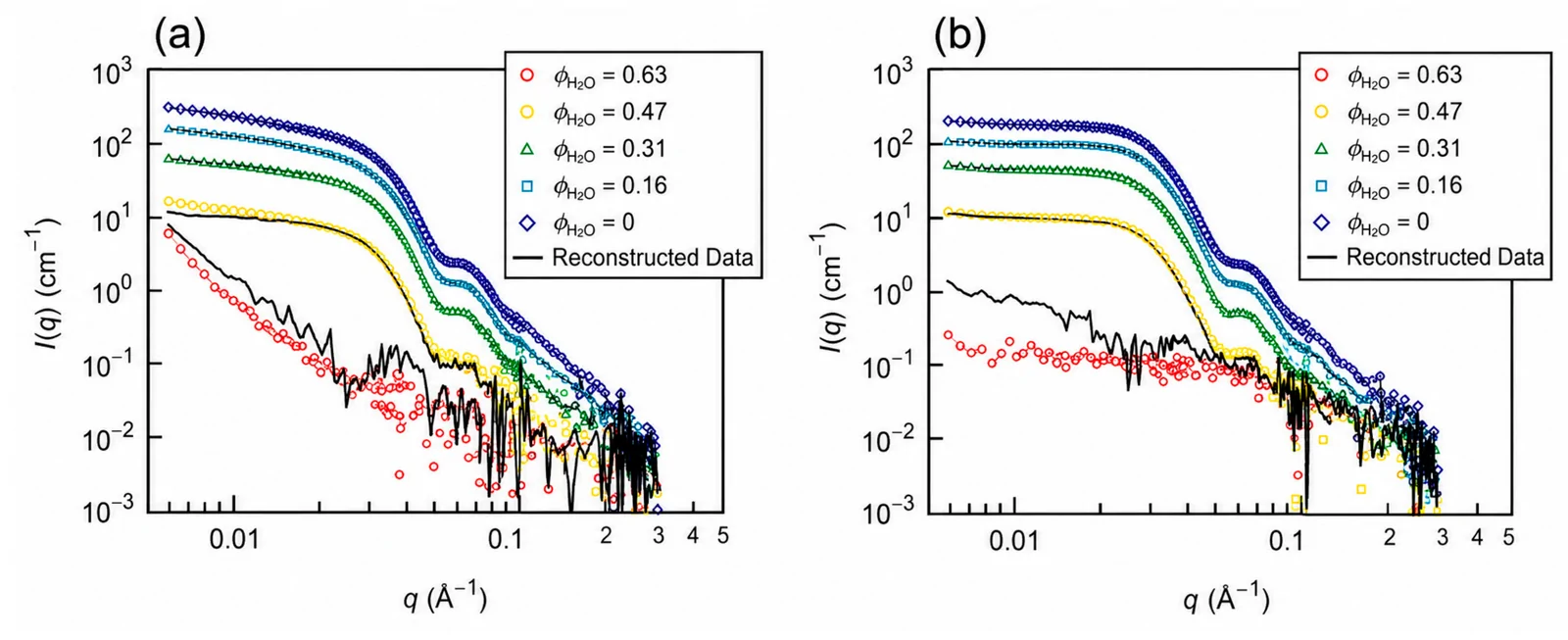
SANS profiles of the (**a**) silica/PAAm and (**b**) silica/PEO systems at ϕp = 0.05 measured using different H_2_O/D_2_O solvent contrast ratios. The solid lines represent the reconstructed scattering functions obtained from the partial scattering functions ICC, ICP, and IPP, showing excellent agreement with the experimental profiles and confirming the successful application of solvent contrast variation by Kusano et al. [186].

SAXS and SANS are able to correlate structural changes with adsorption behavior under realistic conditions. Contrast variation SANS enables quantitative determination of the polymer between bulk and confined phases, providing insight into the thermodynamics of the adsorption-driven accumulation process [185]. Contrast-matching SANS is used to suppress scattering from solvent-filled pores and thereby isolate the signal from the solid matrix, enabling quantitative determination of Q-dependent pore accessibility and comparative analysis of pore structure in porous materials (Figure 6) [186]. Contrast variation SANS (CV-SANS), achieved by tuning the H_2_O/D_2_O ratio, was used to decompose the scattering into self- and cross-terms, enabling direct identification of polymer adsorption; this revealed strong PAAm adsorption forming a thick (~186 Å) polymer shell and a positive silica–polymer cross-term indicative of bridging, whereas PEO showed a weak or negative cross-term consistent with negligible adsorption and depletion-dominated interactions (Figure 7) [187].

By combining SAXS and SANS measurements, a differentiation between mechanisms, such as bridging and depletion in complex colloidal dispersions, is allowed by linking interparticle potentials with adsorption structure. Moreover, by combining SAXS with Size Exclusion Chromatography (SEC-SAXS), this capability is extended by resolving complexation in solution, leading to discrimination between free and bound species, thus providing quasi-operando insight into adsorption processes in soft matter [189].

**Figure 8:**
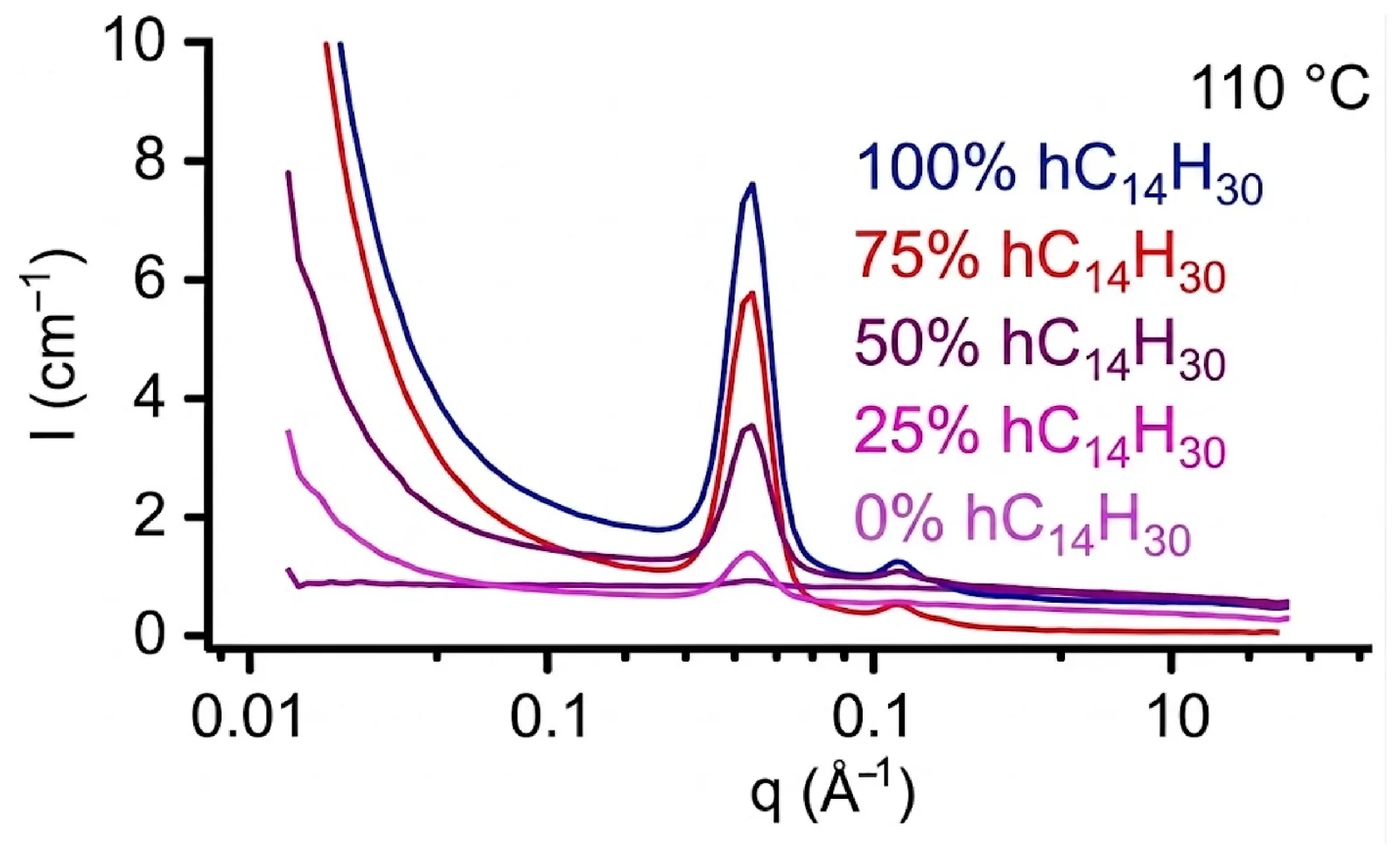
Contrast variation SANS of SBA-15 using dC14-hC14 mixtures at 110 °C. These data were used to determine the contrast match point for the SBA-15 as studied by Fawaz Motolani et al. [193].

In porous or confined systems, in situ scattering techniques can track adsorption-related processes, such as pore filling, ligand spacing and structural evolution. Fawaz Motolani et al. used contrast-variation SANS measurements of SBA-15 with dC14/hC14 mixtures at 110 °C to determine the contrast-matching point of the SBA-15 silica framework, highlighting the capability of SANS to selectively isolate different phases in complex confined systems (Figure 8) [190]. SAXS can similarly monitor polymer adsorption in mesoporous structures through time-resolved contrast changes during pore filling, providing kinetic insight into transport and uptake mechanisms. In addition, SANS can quantify polymer adsorption indirectly by tracking depletion of species in the bulk phase after exposure to adsorbing surfaces [190]. In porous polymer adsorbents, SAXS also captures preservation or evolution of long-range order during adsorption–desorption cycles [191]. Specifically, SAXS can monitor polymer adsorption in mesoporous structures through time-resolved contrast changes during pore filling, offering kinetic insight into transport and uptake mechanisms [192].

**Figure 9:**
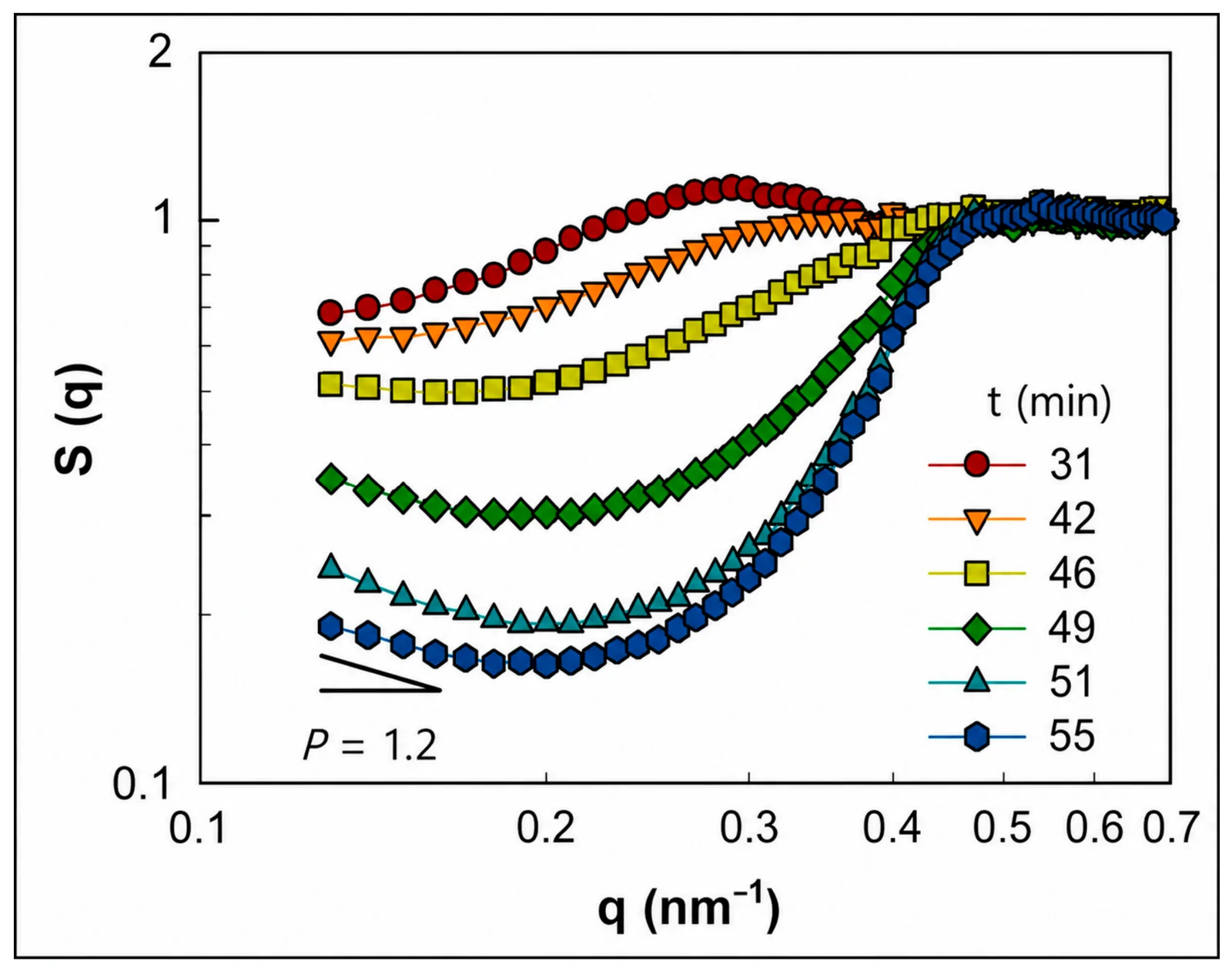
SAXS analysis spectra of silica-PVA film showing the evolution of drying in the range of drying time as studied by Sunhyung Kim et al. for 33 to 55 min [204].

Importantly, SAXS can resolve adsorption-driven structural changes in both bulk and interfacial polymer systems, providing in situ access to nanoscale density variations and interfacial heterogeneity in membranes, which correlate with adsorption, swelling and transport properties [192]. Sunhyung Kim et al. reported SAXS analysis of silica–PVA films, showing the evolution of structural changes during drying over the time range of 33 to 55 min (Figure 9) [194]. In hydrogels and nanocomposites, adsorption can include swelling, intervention or restructuring of polymer networks that can be observed as changes in scattering profiles and interlayer spacing [195]. More generally, SAXS allows direct correlation between adsorption processes and nanoscale reorganization, revealing whether species are trapped, interact with, or restructure the adsorbent [180].

Adsorption effects are often manifested through measurable changes in scattering form factors and structure factors. For instance, polymer adsorption onto fibrous or colloidal structures can be quantified through systematic changes in effective particle size, radius and shape distribution [196,197]. In drying or film-forming systems, increasing adsorption density can suppress structure factor peaks and alter fractal scattering, directly linking adsorption to evolving cluster formation and microstructure development [198]. SAXS also enables identification of adsorption regimes in colloidal systems through changes in interparticle correlations and porous behavior, providing indirect but robust evidence of adsorption-driven assembly [199,200].

However, SAXS and SANS interpretation remains model-dependent and typically requires complementary techniques for full mechanistic resolution [201,202]. While it can track adsorption-induced structural evolution in real time, it does not directly measure molecular binding events, thus making surface-sensitive methods necessary for full validation. Nevertheless, when combined with thermodynamics or surface-sensitive techniques, SAXS and SANS provide a powerful framework linking nanoscale structure to adsorption behavior [203].

Finally, in operando applications, SAXS enables real-time monitoring of structural evolution during adsorption, processing or environmental changes, capturing size, anisotropy and fractal characteristics of evolving systems [190]. It can distinguish between pore filling and interaction mechanisms in adsorption processes by analyzing scattering model changes and structural parameters [195]. Across polymer, colloidal and hybrid systems, SAXS is a key tool for connecting nanoscale structural dynamics with macroscopic adsorption performance [190,191].

### 5.2. Carbon-Based Materials

The increasing demand for energy storage, gas separation, water purification and heterogeneous catalysis has led to the development of advanced porous carbon materials. Understanding their behavior under realistic operating conditions. For this reason, SAXS and SANS have been used widely for the characterization of carbon-based materials, as they have the capability to reveal the nanoscale structure of porous materials [205]. In general, carbon-based materials belong to a huge range of structural organizations, from highly ordered model systems to disordered industrial carbons [206]. As far as in situ and operando use of SAS techniques is concerned, each type of carbon-based structure has been successfully investigated, especially for adsorption phenomena.

Specifically, hierarchical carbon nanospheres with interconnected macro-, meso-, and micropores are an excellent example for studying sequential gas adsorption because the different pore regimes give distinct scattering signatures [207]. Also, there are MOFs such as Ni_3_(HITP)_2_, although not purely carbonaceous, that possess ordered nanopores that tend to imitate the electrical conductivity of carbons and thus have been used as model supercapacitor electrodes in order to resolve ion-specific charge-storage mechanisms [208]. Furthermore, operando wide-angle neutron scattering has been used for hard carbons (non-graphitisable disordered carbons) in order to track both graphene-layer expansion and nanopore filling at the same time as they are employed as anodes in sodium-ion batteries [194,209]. Moreover, plenty of natural carbonaceous materials are used in in situ and operando contrast-matching SANS with the aim of quantifying gas densification in micropores and of distinguishing open from closed porosity under realistic conditions such as pressure and temperature. For instance, coals and shales, despite their structural heterogeneity, have been used widely for this purpose. Contrast-matching SANS using deuterated fluids (D_2_O, CD_4_) and pressure-tuned gas scattering was employed by Zhao Y. et al. to selectively suppress scattering from specific pore populations, enabling the quantification of closed porosity and revealing non-bulk adsorbed gas densities and hierarchical pore filling mechanisms during adsorption [205].

**Figure 10:**
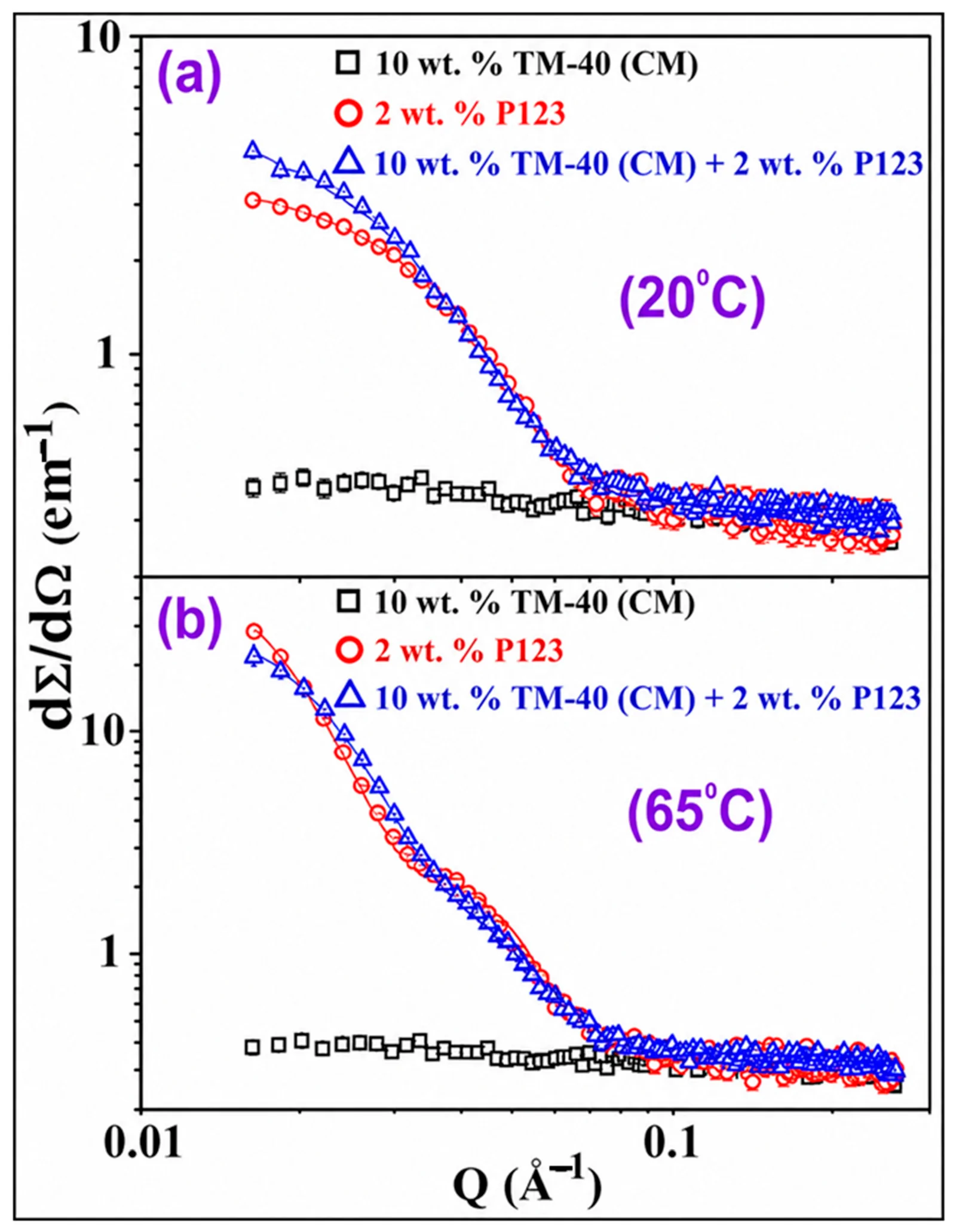
SANS plots and corresponding fits for 10 wt % TM-40 with 2 wt % Pluronic P123 in a 63 vol % D_2_O and 37 vol % H_2_O mixed solvent at (**a**) 20 °C and (**b**) 65 °C, measured under silica contrast-matching (CM) conditions to suppress scattering from the silica nanoparticles. This enables isolation of the polymer-related scattering and analysis of micelle structure and interparticle interactions, including adsorption and association behavior in the mixed system [211].

Before referring to in situ and operando experiments, it is significant to mention contrast-matching methods, which are primarily used to distinguish open from closed porosity in carbon-based materials. Contrast-matching SANS is used in adsorption studies to suppress the scattering contribution of one component, allowing selective observation of the adsorbate distribution within pores or on surfaces (Figure 10) [193]. Contrast-matching SANS is used to suppress specific scattering contributions and thereby enable quantitative analysis of pore accessibility and adsorption behavior in porous systems by distinguishing between bulk and adsorbed phases within the pore network [188].

In the SANS analysis, contrast variation was achieved by adsorbing D_2_O into the cryogel so that open pores became contrast-matched with the solid matrix, which suppressed their scattering signal and revealed that the remaining signal originated from closed pores, allowing the authors to quantify a closed-pore fraction of ~1% and a total porosity of ~83.5% [186]. For example, contrast-variant SANS using D_2_O revealed that carbon cryogels have 82.5% open pores and only ~1% closed pores, which are larger and extended with smooth interfaces [210]. Also, using D_2_O to match the carbon SLD, contrast-matching SANS demonstrated that perfluorooctanoic acid (PFOA) forms only small aggregates in microporous activated carbon but grows into large layered structures (radius up to 2.86 nm) in mesoporous carbon with 8 nm pores, proving that mesoporosity enables cooperative PFAS assembly. Lastly, contrast-matching SANS with a 63/37 D_2_O/H_2_O mixture revealed that Pluronic P123 micelles adsorb on silica nanoparticles, forming a patchy shell that drives reversible liquid–liquid phase separation with a lower consolute temperature [193].

Contrast-matching SANS with deuterated methane up to 70 MPa for in situ studying of shales revealed that the adsorbed methane density is lower than bulk density between 10 MPa and the contrast-match point, but becomes higher below 10 MPa and above contrast-match, indicating a non-monotonic evolution of adsorbed phase density [188].

In situ scattering basically monitors every structural change while simultaneously controlling all the external parameters without driving an electrochemical reaction. Mainly, it is important to highlight that in situ synchrotron SAXS during N_2_ and CO_2_ adsorption on anthracite revealed that weak adsorption (N_2_) causes pore compression, while strong adsorption (CO_2_) superimposes adsorption effects, reducing porosity and specific surface area but increasing average pore diameter. In this way, the positive deviation slope correlated positively with gas sorbability, and fractal analysis showed that CO_2_ adsorption smooths pore surfaces (lower surface fractal dimension) for 20–50 nm pores [212]. As studied by Yixin Zhao et al., in situ SAXS during CO_2_ adsorption at 1–3 MPa on anthracite revealed the decrease in porosity and specific surface area with the simultaneous increase of CO_2_ pressure, while average pore diameter increases too. The scattering intensity ratio revealed that 5 nm pores are most sensitive to high-pressure CO_2_, and irreversible changes after pressure cycling confirm permanent deformation and closure of some nanopores. Another study of in situ contrast-matching SANS examined how CD_4_ and CO_2_ on high-rank anthracite resulted in the reduction in macro-/mesopores, although at the same time increased the micropores by the enhancement of CD_4_ pressure. All these findings collectively indicated that methane densification in micropores where CH_4_ density exceeds liquid methane density proved that adsorption dominates gas storage in micropores; for CO_2_, behavior was more complex due to matrix compression, sorption-induced swelling and pore accessibility effects [213].

An interesting study about in situ synchrotron SAXS on deep (1638 m) versus shallow (420 m) anthracite untangled the complex pore evolution of deep coals as their pore diameter decreases noticeably with CO_2_ at 1 and 3 MPa. As a result, larger structural changes than shallow coal have been noticed, despite shallow coal having higher gas adsorption capacity, thus proving that stress-history (burial depth) dominates adsorption-induced deformation over gas affinity; surface fractal dimension evolution also differs: shallow coal shows a Langmuir-type increase (roughening), while deep coal shows a negative correlation between Ds and average pore diameter, indicating that inner-swelling due to higher ash content is the controlling mechanism [214]. Furthermore, time-resolved GISAXS with remotely controlled surfactant spreading revealed that CTAB acts as a “modifier” (attaching to silica nanoparticles) rather than a “promoter”, with nanoparticle adsorption only beginning after the spread CTAB layer partially dissolves; the surface excess followed a two-step process (diffusion-controlled then first-order kinetics) where the final monolayer density and kinetic constant were primarily controlled by ionic strength rather than surfactant concentration [215].

**Figure 11:**
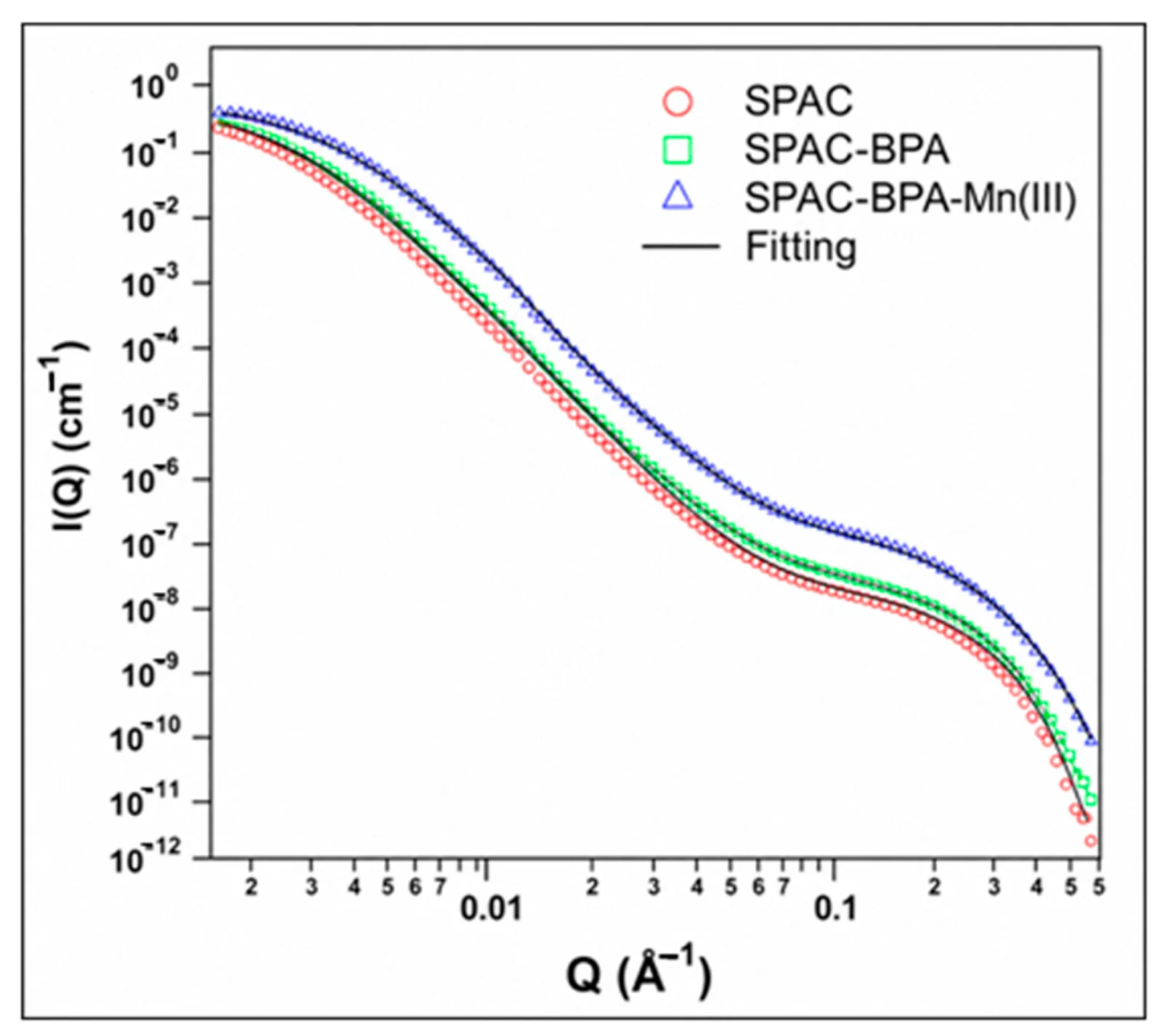
SANS data obtained by Sun Y. et al. Background-corrected SANS profiles of dried SPAC, dried SPAC loaded with adsorbed deuterated BPA, and dried SPAC with adsorbed deuterated BPA following Mn(III)-induced degradation [217].

Moving on to the latest advances in operando studies about SAXS and SANS for carbon-based electrodes, there are experiments that combine scattering with simultaneous electrochemical operation, which offers the opportunity to directly correlate structural changes and functional performance [216]. For instance, Sun Y. et al. reported background-corrected SANS profiles comparing dried SPAC, dried SPAC loaded with adsorbed deuterated BPA, and dried SPAC after Mn(III)-induced degradation of the adsorbed deuterated BPA, highlighting structural changes associated with adsorption and subsequent chemical transformation (Figure 11) [217]. Experiments in which operando SANS tracked CO_2_ adsorption across hierarchical macro–meso–micropores in carbon nanospheres, revealed that CO_2_ first fills micropores to a liquid-like density (correlation peak at Q ≈ 1.65 Å^−1^), followed by capillary condensation in mesopores and eventual grain densification. As a result, the non-monotonic contrast variation in mesopores with pressure that was applied directly proved stepwise transport from macropores to mesopores and eventually to micropores. Operando SANS contrast variation measurements, achieved by varying the CO_2_ pressure, further revealed liquid-like densification of CO_2_ within interconnected micropores and demonstrated sequential diffusion from macro- to meso- to micropores in the M3C structure [207]. Operando SAXS with a core–shell cylindrical form factor model revealed that TFSI^−^ anions form a ~0.25 nm thick immobilized layer near the pore walls of the MOF Ni_3_(HITP)_2_ via F···H–N interactions, creating an electron-dense shell that remains unchanged under applied voltage, forcing a cation-dominated charge storage mechanism (solely Na^+^ adsorption/desorption); the absence of voltage-dependent changes in the form factor and X-ray transmission proves that anions are pinned, with subtle reversible Bragg peak shifts (~0.1%) indicating adsorption-induced pore breathing without ion intercalation [208].

Another significant use of operando SANS and WANS techniques is to study hard carbon anodes for sodium-ion batteries. The structural changes across a very wide range of scales from 0.05 to 50 nm^−1^ have been studied, and it was discovered that sodium ions (Na^+^) do not randomly fill the carbon. Instead, the ions follow a strict, sequential storage process. In the first phase (the “slope region”), Na^+^ inserts itself between the disordered graphene layers of the carbon. This insertion forces the interlayer distance to expand from ~0.335 nm to ~0.389 nm and increases structural disorder. In the second phase (the “plateau region”), the remaining Na^+^ fills the smallest nanopores. This pore-filling causes a decrease in small-angle scattering intensity because the contrast between the solid carbon and the empty pores is reduced. The researchers used the Debye–Bueche model to confirm that this intensity drop continuously tracks pore filling. At the same time, the shift in the graphene layer peak quantitatively measured that ~28% of the interlayers were filled at full charge. Together, these results prove the complete sequence: Na^+^ first occupies edge and defect sites, then inserts between graphene layers, and finally fills internal nanopores [194]. The same sequential mechanism was confirmed in a second independent operando SANS/WANS study on hard carbon, providing clear evidence that intercalation occurs mainly during the sloping region and pore filling during the plateau region, demonstrating the power of simultaneous small- and wide-angle neutron scattering to unravel complex, multiscale storage mechanisms in disordered carbons [209].

### 5.3. Biological Systems

Biological adsorption processes play a pivotal role in medicine, pharmaceuticals and biotechnology [218]. For the investigation of these systems, both SAXS and SANS are ideal in that they allow for the monitoring of adsorption kinetics and the conformational rearrangements that occur during the course of various sample interactions and surface configuration, without destroying the sample [219]. Contrast-matching SANS experiments demonstrated that selectively suppressing the scattering of the solid matrix enables direct observation of adsorbate distribution, revealing adsorption-driven structural organization and pore filling in porous carbon and nanoparticle systems [220]. Furthermore, in situ techniques such as these, unlike ex situ analyses, allow for the recording and investigation of dynamic adsorption processes in real time, while providing additional important structural information at the nanoscale [221]. The ability of SAXS and SANS to investigate adsorption processes while preserving hydration and molecular mobility within the system is the greatest advantage of choosing these techniques for the study of biological systems.

Small-angle scattering techniques are important sources of information and insights into the adsorption of proteins and biomolecular layers near interfaces formed by solid–liquid and liquid–liquid interactions [209]. The adsorption of proteins onto materials has been a field of study for many years and primarily concerns charged surfaces, whether hydrophilic or hydrophobic, as well as biological materials. Regardless of the material, numerous applications have emerged, including biosensors, energy and biofuel production, biological implants, pollutant degradation for environmental purposes, as well as food production and improvement. Protein adsorption is based on numerous interactions, such as hydrophobic forces, van der Waals interactions, covalent bonds (primarily disulfide bridges), hydrogen bonds, electrostatic interactions and stereochemical effects [222].

In situ techniques such as SAXS and SANS have been demonstrated in published scientific studies to be powerful tools for elucidating the adsorption mechanisms of biomolecules, particularly proteins, at scales ranging from 1 to 100 nm [223]. SAXS measurements allow for the determination of protein size, the identification of aggregates and the study of structural organization during the adsorption process, while important parameters such as pair-distance distributions, particle morphology and the radius of gyration (Rg) are directly extracted from the scattering profiles [224]. SANS provides even more useful data than SAXS, because the variation in neutron contrast is exploited for the selective imaging and observation of protein molecules through isotopic substitution, which is particularly useful in cases where X-ray contrast is insufficient in hydrated systems [225].

Biological interfaces are dynamic and structural environments with complex behavior, and they play a role in adsorption processes ranging from the normal physiology of membranes to nanomedicine. Cell membranes, biofilms, lipid bilayers, and protein substrates constitute biological interfaces that exhibit significant heterogeneity compared to inorganic surfaces [226].

**Figure 12:**
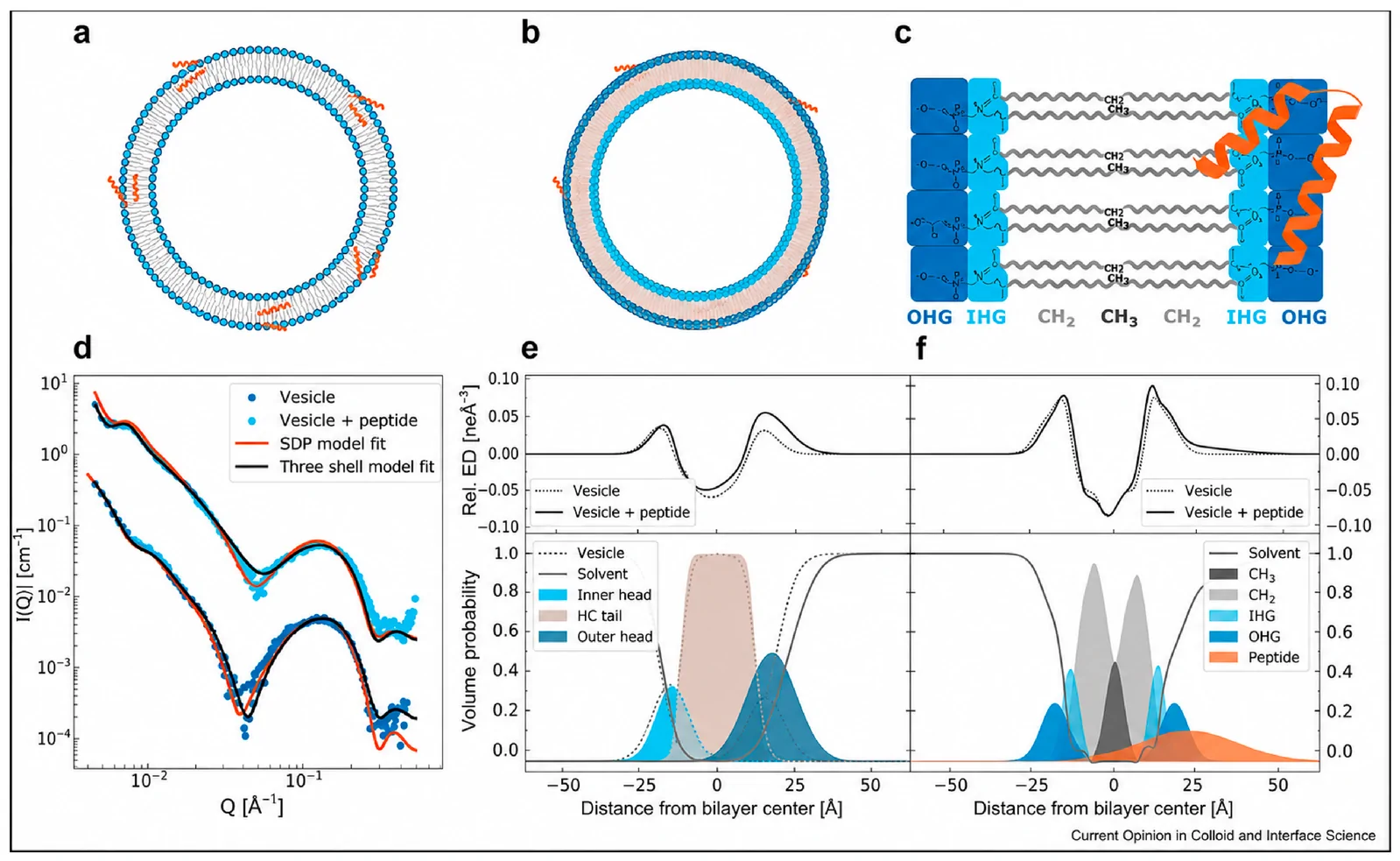
(**a**–**c**) Schematic representations of a vesicle system and bilayer structural models, including peptide insertion at the membrane interface and the partitioning of the bilayer into quasi-molecular components used in the SDP framework. (**d**) Small-angle scattering data from unilamellar vesicles composed of DMPC/DMPG (80:20 mol%) in aqueous solution, with and without Cecropin A (peptide:lipid ratio 1:10). The peptide-containing curve is scaled by a factor of 10 for clarity. Experimental scattering profiles (symbols) are fitted using both the SDP model (red lines) and the three-shell model (gray lines), showing good overall agreement across the measured q-range. (**e**,**f**) Structural parameters derived from model fitting, including electron density and corresponding volume fraction profiles, illustrating bilayer structural rearrangements upon peptide incorporation, particularly in the interfacial headgroup region [226].

Numerous studies have experimentally utilized SAXS and SANS techniques to investigate adsorption information, with the adsorption of peptides and proteins onto bilayers being at the forefront. Nielsen et al. investigated and highlighted the importance of small-angle scattering techniques in peptide-membrane interactions, demonstrating that both SAXS and SANS can analyze structural transformations, such as membrane thinning and changes in curvature during adsorption. In their work, they further emphasized that adsorption at biological interfaces occurs through transient states of non-equilibrium that cannot be studied using ex situ characterization techniques (Figure 12) [227]. In their study, Mahieu and Gabel demonstrated that distinguishing between lipid bilayers, solvent molecules, and proteins in complex systems is possible using selective deuteration strategies, directly confirming that SANS is a highly useful tool in the study of biological interfaces, thanks to the selective imaging capabilities mentioned above [226].

In addition to membrane systems, both SAXS and SANS are also widely used in the study of adsorption involving nanoparticles and the composition of biomolecular crowns [223]. SAXS studies show that the characterization of protein corollas is possible, as is the monitoring of biomolecule adsorption onto nanoparticles during formation. Biomolecular adsorption can significantly affect the size and properties of nanoparticles, as well as their stability over time [228].

Small-angle X-ray scattering (SAXS) and small-angle neutron scattering (SANS) are suitable techniques for studying conformational changes that occur in biological systems, as they provide structural characterization analyses in a hydrated environment without the need for crystallization. Dynamic transition analyses are also possible in real time and cover analyses of the system’s molecular state and molecular flexibility. One of the main and most important applications of SAXS in the study of biological adsorption is the analysis of protein unfolding induced by the adsorption process. Such analyses are interpreted using Kratky plots and scattering profiles in the Guiner region [62].

Operando measurements are also particularly useful in the study of rapid adsorption, which involves molecules such as peptides and protein membranes. In fact, Trewhella et al. emphasized in their research that SAXS enabled the analytical prediction of transitional conformations, which occurred on timescales of milliseconds to seconds, thereby elucidating structural states that are out of equilibrium and cannot be recorded using ex situ methods [223].

Research also uses SAXS and SANS to study the rearrangements of polymeric systems, such as those based on hydrogels and other polymeric networks, during protein binding, thereby monitoring changes in the lattice size and local rearrangements [222].

### 5.4. Ceramic Materials

The use of SAS techniques in order to study adsorption phenomena in ceramic materials remains underdeveloped in the literature, especially regarding in situ and operando experiments. Nevertheless, there are different categories of ceramic materials used as model adsorbents for in situ and operando SAS investigations of adsorption phenomena. Some examples are mesoporous silicas, organosilicas and transition metal oxide nanoparticles, which possess high surface area, adaptable pore architectures and well-defined surface chemistry that make them suitable model adsorbents for in situ and operando SAS investigations of adsorption phenomena [229,230,231].

To date, the majority of studies using SAS techniques on these materials have been conducted ex situ. The techniques are primarily used to characterize structure before and after adsorption rather than during the process. Nevertheless, these experiments have successfully revealed clay exfoliation [231] and interlayer expansion after dye or Cr(III) uptake, quantified surface fractal dimension in zeolites following Cs^+^ and Sr^2+^ exchange [232], validated preserved hexagonal mesostructure in SBA-15 after functionalization [233], distinguished ordered from disordered siliceous foams [213], correlated MOF-polymer interfacial interactions with CO_2_ plasticization resistance [234] and revealed bimodal pore structures in δ-MnO_2_ [235]. As discussed in Section 2.1.3, the deviation of the Porod slope from −4 indicates fractal surface roughness, with the fractal dimension D = 6 − α [1,5]. While not capturing dynamic processes, these studies unequivocally demonstrate SAS sensitivity to pore size, surface roughness [236,237], layer spacing, and fractal dimension. Small-angle scattering techniques (SAXS/SANS) provide a powerful way to connect nanoscale structure with adsorption performance, even though they do not directly measure adsorption at the solid–liquid interface. In systems such as ionic liquids on metal surfaces, SAXS reveals the formation of alkyl-chain-dependent reverse micelles in the bulk phase, where organized or disordered aggregates regulate how molecules are transported toward steel and subsequently form protective adsorbed layers via electrostatic and π–π interactions. In mesoporous materials, SAXS/SANS and USANS further resolve key structural descriptors such as pore symmetry, lattice spacing, domain size, and particle morphology, which are essential for understanding heterogeneous metal ion adsorption behavior and capacity differences [238]. In addition, scattering contrast analysis enables indirect quantification of adsorption within confined pores by tracking pore filling, distinguishing between partial and complete occupation and, in some cases, identifying preferential uptake in multicomponent systems. Overall, these techniques provide a multiscale picture linking solution-phase organization, pore architecture, and adsorption efficiency, allowing discrimination between kinetic and equilibrium-controlled uptake processes [230]. All these parameters can essentially govern adsorption capacity and kinetics in a direct way.

Although only a limited number of in situ and operando SAS studies on ceramic adsorbents have been reported to date, these few investigations are remarkably enlightening and highly promising, leading the way for the next generation of adsorption experiments. Encouragingly, the studies that already exist illustrate the unique insights available. Specifically, in situ SANS during CO_2_ adsorption on SBA-15 up to 55 bar has revealed hierarchical pore filling, where a liquid-like adsorbed phase first occupied micropores within the mesopore walls before mesopore condensation [239]. Also, the non-monotonic variation in the peak intensity has provided direct evidence of the evolution of the adsorbed phase density. In situ SANS contrast variation during CO_2_ adsorption in SBA-15 showed that increasing CO_2_ pressure altered the scattering contrast between the silica walls and pore fluid, revealing densely adsorbed CO_2_ in micropores and suggesting the formation of an adsorbed liquid-like CO_2_ phase along the cylindrical mesopore walls. The contrast with the silica wall was reduced because of the altering scattering length density of the pore fluid. That resulted in the simultaneous tracking of both pore filling sequences and the physical state of the confined adsorbate [239].

Another remarkable example is represented by an in situ SAXS and SANS study with contrast-matching adsorbates that enabled real-time differentiation between homogeneous and phase-separated organic functionalities in PMO ceramics during active physisorption. In this system, contrast matching and contrast variation were used to selectively balance the scattering length density of the pore fluid against the silica-based framework, enabling isolation of adsorption-driven structural changes from background matrix contributions. This approach also avoided the framework contraction artifact that plagues conventional N_2_ physisorption at 77 K [222]. Moreover, a flow-cell in situ SAXS configuration monitored SrTiO_3_ nanoparticles during methylene blue photodegradation. In this experiment, changes in the radius of gyration in the Guinier region directly revealed that vacancy-rich surfaces adsorb more molecules under light. The in situ SAXS signal correlated with macroscopic performance, showing that vacancy-rich samples achieved 39% degradation efficiency versus only 14% for non-irradiated samples [229]. Methodologically, while tracking the intensity of the pore structure Bragg peak during in situ SAXS of polymer melt infiltration into mesoporous silica, a quantitative measure of pore filling kinetics was provided. This approach is directly transferable to studying gas capillary condensation and vapor adsorption in ceramic adsorbents [213].

Although studies involving conventional gas or solute adsorption on ceramics with in situ SAS remain limited compared to the wealth of ex situ characterization, the field shows clear promise. The superior low-angle resolution of SAXS for studying swelling clays without preferred orientation artifacts is particularly notable. The ability of SAXS to quantify pore morphology parameters that govern adsorption further supports its potential for in situ applications [229,235]. A representative benchmark example demonstrating the quantitative agreement between adsorption-derived and scattering-derived pore structure is provided by worm-like mesoporous silica systems [239]. Nitrogen adsorption (N_2_ and Ar physisorption analyzed via NLDFT) and small-angle neutron scattering (SANS) provide consistent but complementary descriptions of pore structure in worm-like mesoporous silicas. While adsorption isotherms yield mesopore size distributions and micropore volumes through model-dependent analysis of capillary condensation and filling behavior, SANS directly probes the spatial arrangement of the pore network and its evolution during adsorption in a more physically continuous manner. In the SE3030 silica system, both techniques converge on a mesopore diameter of approximately 9.5 nm, demonstrating strong agreement for the mesoporous regime. Importantly, SANS additionally captures structural information inaccessible to adsorption alone, such as the organization of the pore network and changes in scattering with progressive pore filling, while also revealing limitations of adsorption-based interpretation in disordered systems where hysteresis and metastability complicate pore size assignment. Together, the results show that SANS provides a robust independent structural validation of adsorption-derived pore size distributions, while offering enhanced sensitivity to pore connectivity and hierarchical porosity [240].

Future development of dedicated in situ cells for controlled atmosphere, flow-through liquid exposure, and temperature/pressure-programmed adsorption appears promising for transforming ex situ SAS validation into operando mechanistic understanding.

All the information regarding SAXS/SANS and adsorption is summarized in Table 4 and Table 5 below.

## 6. Machine Learning and Artificial Intelligence in Small-Angle X-Ray and Neutron Scattering Analysis

Machine learning is increasingly being integrated into SAS, with the aim of enhancing the techniques and their results, as it facilitates direct comparability between experimental data and theoretical models with relatively little processing [211]. Recent developments in machine learning and artificial intelligence have significantly influenced the interpretation of data derived from SAXS and SANS [243,244]. The main advantage is the ability to generate large volumes of synthetic SAXS data using molecular dynamics, Monte Carlo simulations and geometric models [211]. Various machine learning methods have also been evaluated, such as logistic regression, support vector machines, ridge regression, and random forest algorithms, with the latter showing the best comparative performance, with a classification accuracy of 80% [244]. One-dimensional scattering curves I(q) which were created for a total of seven scattering models and are used in the study of soft materials, showed that the random forest model achieves the highest accuracy and this, in turn, showed improvements with the increase in training data sets, requiring a one million training data sets, achieving a classification accuracy of approximately 90% [244].

Artificial intelligence and machine learning are also extremely useful in predicting structural properties from SAS spectra, in which the aforementioned machine learning models are trained to predict important structural parameters, such as the radius of gyration and the maximum particle dimension [211]. Using this technique, a machine learning framework has been developed that is specifically designed to extract structural information directly from SAXS patterns, using data mining and machine learning concepts to predict structural parameters such as maximum particle size, molecular mass, and radius of gyration [245]. For example, by compressing SAXS patterns into a low-dimensional parameter space, such as k-nearest-neighbor classification, different geometric shapes can be identified without fitting traditional models [211].

When creating large simulated datasets containing hundreds of thousands of scattering patterns, k-nearest neighbor algorithms and k-d-tree searches were used to perform classifications and predictions, achieving high classification accuracy, specifically 96.5%, while the impressive result was that a clear distinction was made between compact, flat, hollow and random chains of macromolecular structures. This method converts the data to the dimensionless Kratky scale and calculates the normalized Porod. To predict the structural parameters Dmax and molecular mass, a four-dimensional feature space has been developed that combines the three dimensions V’ and Rg and estimates the parameters using normalized inverse Euclidean distance as weights [245,246].

Machine learning also facilitates the extraction of cross-sectional interactions. Some of these interactions are the force field parameters derived from SAS analyses and comparisons of experiments and simulations [247]. A machine learning framework has been developed, based on Gaussian process regression, for the inverse determination of polymer configuration parameters from scattering intensity data, without relying on predefined analytical scattering models, using Monte Carlo simulations and creating libraries of S(q) factors as training datasets [248,249]. Machine learning also finds applications in SAXS and SANS techniques through inverse modeling, where microscopic and energy-related parameters of scattering data are extracted, by combining Monte Carlo simulations with experimental data derived from these techniques and generates datasets that enable principal component analysis and dimensionality reduction through inverse mapping [250]. Inverse mapping is performed via Gaussian process regression, which provides predictions and uncertainties and has been applied to mechanical polymer systems [251,252]. Conventional model analysis cannot handle the vast amount of data and parameters required to account for orientation during the analysis of anisotropic soft materials [253]. This is yet another challenge to which machine learning provides solutions through neural networks [254,255]. For systems with high dispersion and multiple variations in shape and size, a CREASE methodology extracts separate and distinct parameters for both shape and size distributions, using neural networks as surrogate models and interprets high-noise scattering data [256,257,258,259].

Machine learning has made it possible to handle SAXS biological data incredibly quickly, especially with the DATCLASS program linked into ab initio modeling tools [245,260]. Using supervised learning on synthetic SAXS data, feedforward artificial neural networks are utilized to predict the molecular weight and maximum distance from the scattering patterns of folded proteins, disordered proteins and nucleic acid. The authors assert that their neural network-based approach is unique in estimating molecular weight from SAXS data of intrinsically disordered proteins and that it achieves higher accuracy and is less demanding in terms of data quality when compared to established methods for folded proteins and nucleic acids [261,262]. By learning a nonlinear mapping between the scattering intensity profiles I(q) and the underlying structural descriptors of double-stranded RNA, the Extreme Gradient Boosting method, which was trained on sizable datasets produced from molecular dynamics simulations, has also been used to interpret small-angle and wide-angle X-ray scattering data [217,263].

Additional applications in SAS data collection and processing include the use of B-spline Gaussian mixture models to predict high-angle scattering from limited data, thereby reducing measurement time and convolutional neural networks for achieving super-resolution reconstruction of anisotropic scattering patterns [211,264]. A machine learning framework specifically addresses the challenge of sparse or highly noisy data, allowing for successful extraction of structural parameters and hidden information from data collected with significantly shorter exposure times by integrating ML directly with the physical principles governing scattering. Neural networks [265] trained on extensive datasets generated via synthetic simulations recognize distinct scattering patterns corresponding to various nanostructures, and when presented with sparse, noisy or incomplete experimental SANS/SAXS curves, the trained networks can reconstruct the full scattering profile, filter out experimental noise and accurately predict structural parameters [266,267,268]. The Bayesian approach allows the system to seamlessly combine prior knowledge with newly acquired imperfect experimental data through probabilistic frameworks such as Gaussian Process Regression, quantifying error bars and uncertainty bounds to ensure predictions remain scientifically rigorous and interpretable. An uncertainty-aware machine learning framework for SAXS analysis in autonomous experimentation trains a random forest regression model on 100,000 synthetic SAXS curves simulating polydisperse spherical nanoparticles with realistic experimental background noise and by feeding normalized 1D SAXS intensity profiles into the model, it directly and instantaneously predicts nanoparticle radius, size polydispersity and background parameters. The integrated uncertainty metric acts as an automated filter, flagging high-confidence data that match the trained structural model to prevent the autonomous system from trying to fit incorrect physical models to bad or mismatched data [268,269].

The SCAN tool, an open-source machine learning tool designed to assist SAXS analysts in structural model selection, generates training data using SasView software simulating SAXS curves for eight geometric models and three statistical models, implementing nine classifiers including Random Forest, XGBoost Classifier, Multi-layer Perceptron and stacked ensemble models [270]. Evaluation using five-fold cross-validation on 3000 simulated curves per model showed that XGBoost and Random Forest classifiers individually achieved accuracies of 95.9% and 95.8%, respectively, using full 500-point q-vectors, while stacked ensembles achieved up to 97.3% accuracy. A machine learning framework for predicting microstructural parameters of disordered porous materials from SAXS data using Gaussian Random Fields as the microstructure model formulated parameter prediction as a nonlinear regression task using XGBoost (gradient boosted trees), chosen for proven accuracy on tabular data and GPU acceleration [271]. Results on test sets showed that the scaling parameter a was most accurately predicted with mean absolute percentage errors ranging from 0.617% to 0.727% across four datasets, while porosity was more challenging with MAPE 1.583–4.190%, and prediction execution time was approximately 2.5 ms per SAXS curve per output on GPU.

Convolutional neural networks have been employed to classify crystal systems directly from diffraction data, with attentive response maps used to visualize which features of the diffraction pattern, such as peak positions and orientations, are most relevant for classification [211]. A one-dimensional convolutional neural network was designed for predicting crystallographic symmetry classes from diffraction patterns, applying 1D convolutions directly to intensity vectors *I*(q), achieving near 100% accuracy for cubic, tetragonal and trigonal classes and 92.65% accuracy for distinguishing monoclinic from triclinic classes, significantly outperforming distance-based algorithms on overlapping classes [272]. For regression of unit cell parameters, random forest regression substantially outperformed non-negative least squares, with coefficients of determination of 0.995 for trigonal, 0.999 for tetragonal, and 0.998 for cubic classes on simulated data, indicating that the structure–scattering relationship is nonlinear. When applied to experimental neutron data of BaTiO_3_, random forest regression achieved mean squared errors on the order of 10^−3^ for cubic, tetragonal and trigonal phases, though performance on experimental data for tetragonal and trigonal classes was less accurate than on simulated data, attributed to sensitivity to background subtraction and the limited feature set of shallow models. The study concludes that while shallow learning models establish a baseline, deeper models are required for general material structure prediction from experimental scattering data. In another approach, SAXSNN, a physics-aware neural network, directly reconstructs real-space morphologies from two-dimensional SAXS patterns without requiring predefined models or large labeled datasets, employing a 2D convolutional encoder–decoder neural network combined with a forward physical model of X-ray scattering [273]. The loss function based on the negative Pearson correlation coefficient is minimized to train the network, operating as an unsupervised learning model that eliminates the need for large manually labeled datasets, with training performed on each single SAXS pattern individually and computation time of about 10 min on a single CPU. For time-resolved SAXS data, an iterative approach retaining network weight parameters from previous iterations can refine reconstruction and provide continuous representation of evolving microstructures. A 3D convolutional autoencoder trained on thousands of 3D protein shapes compresses complex high-dimensional 3D spatial representations into a compact 200-dimensional continuous latent space vector and by restricting the neural network to operate in this optimized latent space, any generated structure remains topologically realistic and structurally sound. To analyze an experimental 1D SAXS curve, the system populates the latent space with candidate structural vectors, and a genetic algorithm automatically evolves and optimizes these vectors, with theoretical SAXS profiles simulated and compared to experimental data until the best-fitting 3D macromolecular model is converged upon.

An end-to-end ML pipeline engineered to analyze 1D SAS intensity curves directly with minimal pre-processing is explicitly built to be data-efficient, learning complex physical representations while requiring significantly less training data than standard models, making it a highly practical tool for deployment directly at beamlines [274]. The network architecture is designed to map and capture hierarchical relationships between similar morphologies, substantially increasing multi-class classification accuracy and the model can extrapolate outside the strict parameter ranges of its training data, with predictions verifiable quickly against physical constraints for live real-time feedback loops. A community benchmark repository containing four specific sub-datasets, including synthetic datasets with 75,000 simulated scattering spectra representing nine different nanoparticle shape models and real-world experimental datasets of 10 physical samples each, was designed to test whether ML models trained on simulations can successfully generalize to real laboratory instruments [275]. The repository allows researchers to train deep neural networks using representation learning, where deep neural networks ingest raw intensity vectors to automatically extract latent geometric features that define distinct types of nanoparticle morphologies, covering nine distinct nanoparticle geometric models for automated model selection. To address the domain-shift problem where models trained on a single instrument configuration generalize poorly to others, the data models two highly popular distinct laboratory instrument setups and includes instrument corrections, including buffer background removal and exposure time normalization.

A study focusing on optimizing the execution of SANS and SAXS experiments via data-driven active learning develops a simulation-based machine learning strategy that relies on active learning where the algorithm chooses its own data [228]. The machine learning algorithm iteratively analyses data points collected so far and mathematically suggests the best target condition for the next sequential measurement to gain maximal structural information with minimal steps, using Bayesian inference via Gaussian Process Regression. The Gaussian Process model calculates a prediction for unmeasured parameter regions along with precise quantification of statistical uncertainty (variance) and the Bayesian framework uses an acquisition function to balance testing areas where a strong structural phase transition is expected against areas where confidence is lowest. As the detector completes a measurement, the live data stream is fed directly into the GPR model, and within seconds the software tells the robotic sample stage exactly what condition to measure next, allowing researchers to map out complete material phase diagrams using a fraction of the data points traditionally required [276]. An intelligent closed-loop software framework acts as an automated expert system linking hardware directly with high-performance computational clusters, handling the complete data pipeline including initial background subtraction, normalization, and structural parameter extraction [277,278]. The automated system checks data frame-by-frame in real time, instantaneously flagging signs of beam-induced radiation damage or sample aggregation, and calculates structural descriptors such as radius of gyration and forward scattering intensity on the fly, determining if the signal-to-noise ratio is sufficient or if the sample has degraded. The automated pipeline then passes data to standard scattering analysis suites for automated model selection, using integrated algorithmic tools to automatically classify the structural state of the macromolecule as globular, elongated, or intrinsically disordered, and automatically triggering ab initio shape determination routines and rigid-body modeling against existing atomic coordinates. Machine learning is also discussed generally for analyzing complex neutron-scattering datasets for pattern recognition and identifying meaningful structural features in large volumetric data, with benzene adsorption in mesoporous silica monitored using neutron scattering to observe structural changes during uptake [279]. Machine learning is increasingly integrated into SAXS workflows to handle large high-throughput datasets by enabling dimensionality reduction, clustering, noise reduction and generative reconstruction of SAXS patterns using methods such as VAEs, cVAEs and hybrid VAE–MLP networks to extract latent structural features and build predictive mappings between processing conditions and resulting nanostructures.

## 7. Challenges

Perhaps the most fundamental challenge is the inherent model dependence of SAS techniques. Unlike imaging methods, SAXS and SANS require fitting to physical models, which becomes particularly problematic when scattering contributions from micropore filling, mesopore condensation, and external adsorption overlap in the same q-range. This ambiguity necessitates complementary techniques for validation [17,18]. Also, changes in scattering intensity during adsorption can originate not only from genuine structural rearrangements but also from contrast variations as adsorbate density changes, so they are called “apparent strain.” Without careful control experiments, misinterpretations of structural evolution remain a genuine risk [92].

Moreover, adsorption spans milliseconds to hours. While synchrotron SAXS achieves millisecond resolution, this comes with poor signal-to-noise ratios. Laboratory instruments provide high-quality data but require minutes or longer, leaving a “kinetic gap” for intermediate timescales that remains difficult to access [29,45,59]. High-pressure gas adsorption experiments (up to 70 MPa) require cells that maintain pressure and temperature while remaining beam-transparent [188]. Operando electrochemical cells must accommodate electrodes and potential control without parasitic scattering [208]. A critical consideration in synchrotron SAXS experiments is radiation damage, particularly during prolonged or repeated X-ray exposures, which can alter sample structure, generate damaged material that accumulates on sample cell walls, and consequently affect scattering profiles, background subtraction, and structural interpretation [280]. To minimize these effects, modern SEC-SAXS methodologies employ strategies such as shutter-controlled exposure, continuous sample flow, and additional cleaning procedures between measurements, thereby improving data reliability and ensuring that observed structural changes reflect the intrinsic behavior of the sample rather than radiation-induced artifacts. Similarly, while contrast variation SANS is a powerful approach based on isotopic substitution, typically through H/D exchange, deuteration may introduce isotope effects that subtly influence hydrogen-bonding interactions, diffusion dynamics, adsorption equilibria, and self-assembly processes [281]. Therefore, appropriate control experiments are often required to confirm that the behavior of the deuterated system remains representative of the corresponding native material and that the obtained structural information is not biased by isotopic substitution.

Isotopic contrast variation in SANS is powerful but expensive and technically demanding. D_2_O-induced aggregation or structural changes can compromise entire contrast series, and validating sample integrity across conditions remains arduous [50,89,90]. Adsorption is inherently multiscale, yet SAS probes primarily the 1–100 nm range [46]. Connecting molecular events to macroscopic responses remains challenging, though combined small- and wide-angle scattering offers promise [109,224].

## 8. Conclusions

This review has critically assessed the application of in situ and operando small-angle X-ray and neutron scattering to adsorption phenomena across soft matter, carbon-based materials, biological systems, and ceramics.

The fundamental advantage of in situ and operando SAS over conventional ex situ characterization lies in its ability to capture dynamic structural evolution during adsorption rather than static before-and-after snapshots. Time-resolved measurements have revealed kinetic pathways, metastable intermediates, and hierarchical pore-filling sequences invisible to traditional methods. Operando approaches, correlating structural changes with adsorption performance, have proven particularly powerful for establishing mechanistic conclusions.

Across material classes, key insights have emerged: polymer adsorption-driven reorganization, sequential sodium storage in hard carbon anodes, protein conformational changes under hydrated conditions, and real-time differentiation of organic functionalities in mesoporous ceramics. These examples demonstrate the unique capabilities of SAS for probing nanoscale adsorption dynamics.

Nevertheless, persistent challenges remain. Data interpretation is model-dependent and ambiguous in complex systems. Distinguishing genuine structural changes from contrast-induced artifacts requires careful experimental design. Timescale limitations create a “kinetic gap” between fast synchrotron and slow laboratory measurements. Deuteration for SANS contrast variation remains expensive and technically demanding.

Regarding future perspectives, advanced contrast manipulation strategies, including triple isotopic substitution and nuclear spin contrast variation, could enable spatial mapping of adsorbate distributions without the complications of deuteration. Machine learning and physics-informed neural networks hold strong potential to accelerate data interpretation and reduce model-dependent ambiguity. Multimodal correlative approaches in which SAS will be combined with spectroscopy, electron microscopy, or microfluidics offer the potential to link chemical, structural, and kinetic information across length scales. Extending these methodologies beyond well-ordered model systems to disordered, polydisperse, and chemically heterogeneous “real-world” adsorbents represents a critical frontier. Addressing these challenges will transform in situ and operando SAS from a specialized technique into a mainstream tool for understanding and engineering adsorption phenomena at the nanoscale.

In summary, in situ and operando SAXS and SANS have fundamentally transformed adsorption research, revealing dynamic mechanisms that ex situ methods cannot capture. Continued methodological development promises to accelerate the rational design of advanced porous materials for gas storage, carbon capture, water purification, and energy storage applications.

## Figures and Tables

**Table 1:** Summarized differences between the structural implications of the scattering exponent [32,78,79,80,81,84,87].

Scattering Exponent	Structural Regime	Physical Meaning at the Pore Interface	Common Material Systems
*α* < 3	Volume (Mass) Fractal	Loosely aggregated, highly porous 3D network clusters; mass scales with a non-integer dimension.	Highly porous polymer networks, swollen hydrogels, and aerogels.
3 < *α* < 4	Surface Fractal	Rough, highly convoluted, and self-similar 2D pore boundaries.	Dry Vycor porous glass, pristine lignite coals, and rough silica matrices.
*α* = 4	Classical Porod Limit	Mathematically sharp, smooth Euclidean interfaces with no boundary gradients.	Unmodified silica controls or pore walls smoothed by thick adsorbed film multilayers.
*α* > 4	Diffuse Interface	Continuous density gradient across a boundary layer of defined physical thickness.	Organo-modified mesoporous molecular sieves (organo-MSU-X) with grafted functional groups.

**Table 2:** Summarized differences between physisorption and chemisorption.

Feature	Physisorption	Chemisorption
Nature of interaction	Weak, physical forces [100,101]	Strong, chemical bonds [100,101]
Energy of interaction	Low [112,113]	High [109,110,111,112]
Reversibility	Reversible (equilibrium) [100,103]	Often irreversible (full surface coverage) [100,103]
Layer formation	Single or multilayer [107,108,109,113]	Single layer only [109,110]
Dependencies	Pressure [108]	Temperature [111]
Activation energy	Usually none or very small [112,113]	Required [109,110,111,112]
Saturation mechanism	Saturation via multilayer condensation or coverage [105,113]	Saturation when all active sites are occupied [108,110,111]
Model	BET [114]	Langmuir [114]

**Table 3:** Summarized differences between gas phase adsorption and liquid phase adsorption.

Parameter	Gas Phase Adsorption	Liquid Phase Adsorption
Definition	Adsorption of gaseous molecules onto a solid surface [114,115]	Adsorption of solutes from a liquid onto a solid surface [120,121]
Phases involved	Gas–solid system [114,115]	Liquid-solid system [120,121]
Driving forces	Mainly physical interactions [117]	Chemical interactions [123]
Selectivity	Generally lower [117]	Higher [122,123]
Role of solvent	No solvent present [120]	Solvent plays a major role [120]
Competition effects	Limited [115]	Strong [120]

**Table 4:** Summarized comparative information about SAXS and SANS in the four categories of materials (Soft matter and polymer system, carbon-based materials, biological systems, ceramic materials) discussed in this review.

Material System	SAXS	SANS
Soft Matter and polymer system	Structural rearrangements caused by adsorption. Monitors properties such as thickness, morphology, and aggregation number [179]. Application in the adsorption of polymers onto mesoporous structures [203]. Study of swelling, membrane heterogeneity, and filling kinetics [194,215].	Quantitative determination of polymer distribution [186]. Determination of adsorption directly via cross-terms [241]. Bridging mechanisms versus depletion mechanisms and indirect adsorption via bulk measurements [187].
Carbon-based materials	Investigation of pore compression, adsorption-induced deformations, and fractal changes [205,212]. In situ SAXS identifies changes in porosity and fractal phenomena during N_2_/CO_2_ adsorption [205]. Operando SAXS investigates ion adsorption within the pores [217].	Distinguishing between open and closed porosity [205]. Contrast and secondary imaging strategies. Quantification of gas density and hierarchical pore filling [205]. Suitable for hidden structures
Biological systems	Measurement of protein size, aggregation, radius of gyration (Rg), and morphology [224]. Used to study protein unfolding, biomolecular crown formation, and adsorption kinetics [242]. Kratky and Guiner analyses are available and utilized in the study of changes caused by adsorption [62].	It is particularly effective in hydrated biological systems due to isotopic contrast [225]. Visual separation of proteins, lipid bilayers, and interfaces [226]. Direct study of complex biological interfaces [226].
Ceramic materials	Application for investigating pore morphology and fractal analysis [229,235]. In situ SAXS monitors pore filling kinetics and photocatalytic adsorption, as well as structural changes in mesoporous ceramics [213].	SANS offers an advantage by varying the contrast during gas adsorption Identification of structural changes caused by adsorption, the sequence of pore filling, and the physical state of the adsorbent [240].

**Table 5:** Models and analysis approaches used to describe adsorption phenomena in SAS (SAXS/SANS) studies.

Model/Analysis Approach	SAS Technique	Adsorption Information Obtained
Guinier model [229]	SAXS/SANS	Radius of gyration (Rg), particle growth/shrinkage during adsorption, aggregate formation
Porod analysis [231,234,235]	SAXS/SANS	Surface roughness, surface fractal dimension, interface evolution during adsorption
Fractal models (mass and surface fractals) [84,205,212]	SAXS/SANS	Adsorption-induced changes in pore morphology, aggregation and surface complexity
Form factor models (sphere, cylinder, core–shell) [196,197]	SAXS/SANS	Adsorbed layer thickness, particle morphology, shell formation around adsorbents
Core–shell cylindrical model [208]	Operando SAXS	Thickness and location of adsorbed ionic layers inside pores
Structure factor analysis [180,181,182]	SAXS/SANS	Interparticle interactions, adsorption-induced ordering, bridging and depletion effects
Contrast variation/contrast matching models [230]	SANS	Direct identification of adsorbed species, pore accessibility, adsorbate distribution, open vs. closed porosity
Bragg peak analysis [213]	SAXS/SANS	Pore filling kinetics, preservation of ordered mesostructure, adsorption-induced lattice changes
Pair-distance distribution function [224]	SAXS	Protein size, shape and conformational changes upon adsorption
Kratky analysis [62]	SAXS	Protein unfolding and conformational rearrangement caused by adsorption
Debye–Bueche model [194,209]	Operando SANS	Quantitative monitoring of nanopore filling during adsorption/storage processes
Hierarchical pore-filling models [239]	SAXS/SANS	Sequential adsorption in micro-, meso- and macropores
Scattering intensity ratio and contrast evolution analysis [212]	SAXS/SANS	Adsorbate density evolution, liquid-like adsorption phases, pore accessibility
Time-resolved SAXS/SANS kinetic analysis [230]	SAXS/SANS	Adsorption rates, transport mechanisms, pore-filling kinetics

## Data Availability

No new data were created or analyzed in this study. Data sharing is not applicable to this article.
